# The pattern of brain-size change in the early evolution of cetaceans

**DOI:** 10.1371/journal.pone.0257803

**Published:** 2021-09-28

**Authors:** David A. Waugh, J. G. M. Thewissen

**Affiliations:** Department of Anatomy and Neurobiology, Northeast Ohio Medical University, Rootstown, Ohio, United States of America; Liverpool John Moores University, UNITED KINGDOM

## Abstract

Most authors have identified two rapid increases in relative brain size (encephalization quotient, EQ) in cetacean evolution: first at the origin of the modern suborders (odontocetes and mysticetes) around the Eocene-Oligocene transition, and a second at the origin of the delphinoid odontocetes during the middle Miocene. We explore how methods used to estimate brain and body mass alter this perceived timing and rate of cetacean EQ evolution. We provide new data on modern mammals (mysticetes, odontocetes, and terrestrial artiodactyls) and show that brain mass and endocranial volume scale allometrically, and that endocranial volume is not a direct proxy for brain mass. We demonstrate that inconsistencies in the methods used to estimate body size across the Eocene-Oligocene boundary have caused a spurious pattern in earlier relative brain size studies. Instead, we employ a single method, using occipital condyle width as a skeletal proxy for body mass using a new dataset of extant cetaceans, to clarify this pattern. We suggest that cetacean relative brain size is most accurately portrayed using EQs based on the scaling coefficients as observed in the closely related terrestrial artiodactyls. Finally, we include additional data for an Eocene whale, raising the sample size of Eocene archaeocetes to seven. Our analysis of fossil cetacean EQ is different from previous works which had shown that a sudden increase in EQ coincided with the origin of odontocetes at the Eocene-Oligocene boundary. Instead, our data show that brain size increased at the origin of basilosaurids, 5 million years before the Eocene-Oligocene transition, and we do not observe a significant increase in relative brain size at the origin of odontocetes.

## Introduction

Cetaceans such as the sperm and killer whales have brains exceeding nine kilograms [1, 2], larger than any other species on the planet, living or extinct. Scaled for body size, the largest cetacean brains are eclipsed only by those of the genus *Homo* [3, 4]. Most studies comparing brain sizes use an index called the encephalization quotient (EQ) to accommodate the fact that animals with larger bodies tend to have proportionally larger brains [2, 4–12]. The EQ value indicates how much larger (EQ>1) or smaller (EQ<1) the brain of the animal being studied is compared to a predicted brain mass for an animal of the same weight (EQ = 1).

The fact that cetacean relative brain sizes approach those of humans has sparked both scientific and public interest in understanding the cetacean brain, with much attention being given to the selection pressures that lead to, and the implications of, the high EQ values observed in the toothed whales (Odontoceti). The high EQs of cetaceans have been attributed to the need of cetaceans for expanded cognitive abilities [10, 13–15]; their complex social structure [16, 17]; their sophisticated echolocation system [7, 18–21]; their diet [22], and even their thermoregulatory needs [23, 24]. Intriguingly, living cetaceans include not only species with high EQ values, particularly the delphinoid odontocetes, but also include species with some of the lowest mammalian EQs such as the balaenid mysticetes [11]. The small relative brain size observed in mysticete cetaceans has been ascribed to the decoupling of brain and body size scaling in conjunction with selection pressure for increased body size [12, 25, 26].

Interest in the high EQ of odontocetes has resulted in a substantial literature exploring both the implications and evolutionary history of cetacean brain size [2, 4, 6–9, 12, 15, 21, 23, 27–32]. Marino et al. [7] published the first large dataset of EQ estimates (including brain and body masses) for fossil cetaceans. This important body of data, sometimes with slight modifications, has become the basis of numerous analyses [8, 9, 12, 21, 23, 31], again, it is clear that Marino’s initial publications on the subject have inspired sustained scholarship on brain size evolution in cetaceans.

In this paper, we re-evaluate the existing data on brain and body size in fossil cetaceans, with a focus on the Eocene, and we present the cetacean data alongside their terrestrial artiodactyl relatives. We believe that some of the initial methods used to estimate brain and body mass, which are needed to calculate EQ, suffer from flaws that have clouded the apparent pattern of fossil cetacean brain size evolution. The purpose of this paper is to build a stronger foundation for the continued study of cetacean brain size evolution. It is our position that the existing data can be improved in three significant ways: 1) the methodology by which brain size is estimated in fossil cetaceans and terrestrial artiodactyls; 2) the manner in which body mass is estimated in fossil cetaceans; 3) and in the scaling factor used to calculate EQs, a point made previously by Boddy et al. [10], and Smaers et al. [31]. In addition, we rectify some erroneous data that have continued to propagate in the literature.

### Estimating brain mass

**Fig 1:**
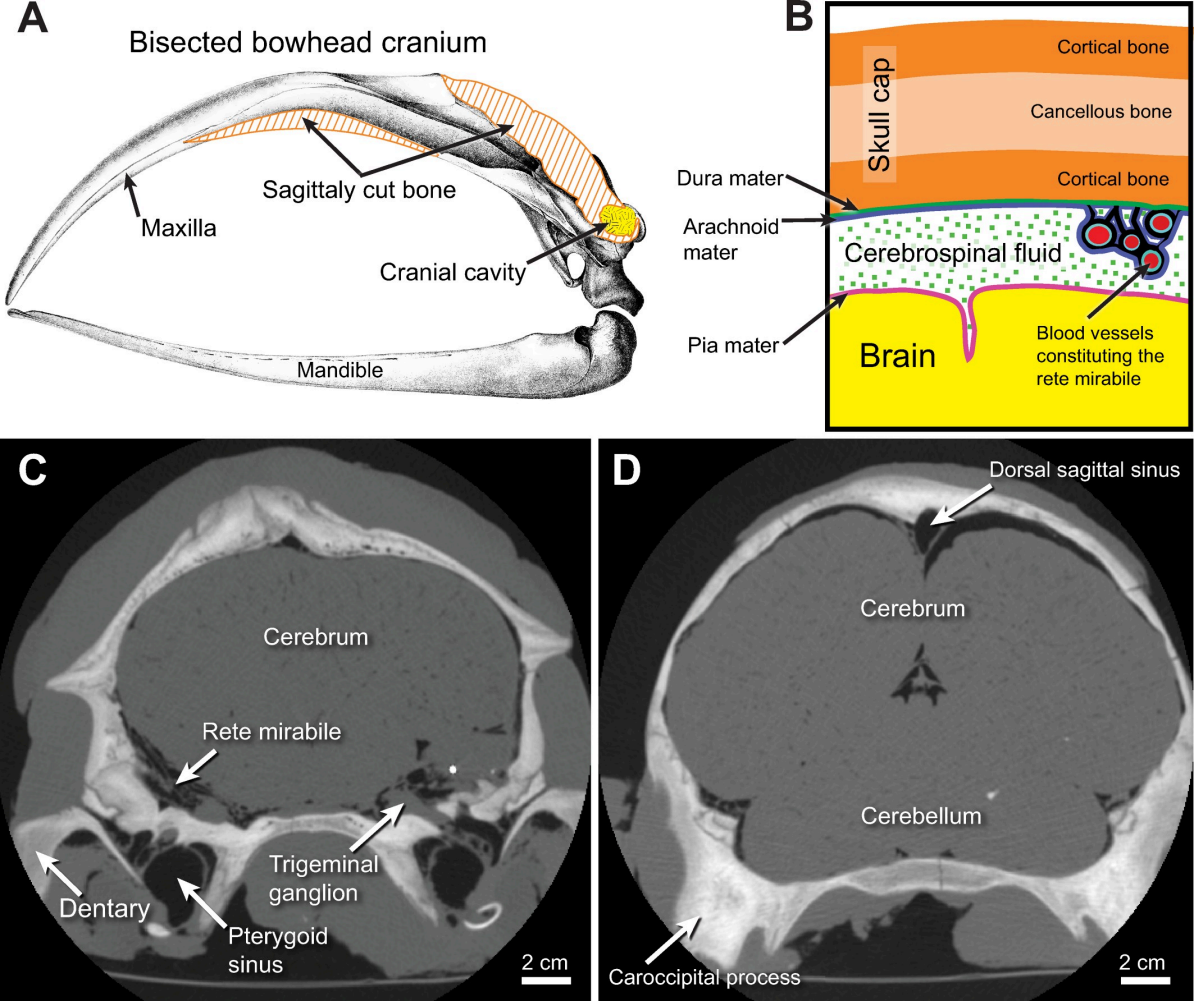
Cranial cavity and anatomy and beluga whale cranial CT scans. (A), line drawing of a bisected bowhead skull (modified Eschricht and Reinhardt [74]); (B), Illustration of cranial cavity components including membranes surrounding the brain; C-D, coronal CT scan slices through the head of a beluga whale (NSB-DWM 2019LDL10); (C) section through the root of the zygomatic arch. (D) more caudal section through the paroccipital process.

Fossil brain mass estimates are derived from the volume of the cranial cavity (Fig 1A) as measured from natural or artificial endocranial cast [5, 33] or, increasingly, from CT scans [14, 34, 35]. To estimate brain mass from endocranial volume, some scientists assume the brain occupies the entire cranial cavity and has a density of 1 g/cm^3^, essentially equating endocranial volume with brain mass [5, 7, 12, 14, 30]. Others [9, 21, 23] have corrected for the specific density of brain tissue: 1.036 g/cm^3^, as measured in humans [36] or 1.04 g/cm^3^, as measured in cetaceans [2].

The volumetric discrepancy between brain and endocranial volume [2, 9, 37, 38] is quantitatively more important than the relatively minor adjustment for brain tissue density. Bowhead whales provide an extreme example as the bowhead brain only occupies approximately 40 percent of the cranial cavity [39] while the remaining 60 percent is filled with adnexa and cerebrospinal fluid. Adnexa in this context refers collectively to the non-brain tissues present within the cranial cavity; specifically, the meninges (which includes the dura mater, arachnoid, and pia mater) (Fig 1B), cranial nerves, dural sinuses, and a collection of arteries and veins which includes the rete mirabile. The rete is a specific structure consisting of a meshwork of blood vessels, which is present in most terrestrial artiodactyls [40–44] and cetaceans [2, 45, 46]. Although a rete is present in all cetaceans, it appears to be disproportionately expanded in extant mysticetes [39, 45]. Although discrepancies between brain and endocranial volume in cetaceans have been observed, they have been generally disregarded, except within the Basilosauridae, an extinct family of cetaceans which are thought to have possessed a significant endocranial rete mirabile [27, 30, 47–50].

Uhen [47, 51] first estimated the rete in the basilosaurid *Dorudon atrox* to have occupied approximately 20% of the cranial cavity volume based on a series of *D*. *atrox* endocasts in which the volume of the rete was approximated using clay. Based on endocast morphology, Gingerich [30] extended this 20% endocranial volumetric rete correction to the basilosaurids *Saghacetus* and *Basilosaurus*, a practice which subsequent authors have followed [7–9, 14], and Marino et al. [14] extended this correction to another basilosaurid: *Zygorhiza kochii*. Although the rete is a component of the adnexa, it must be emphasized that the endocranial volume correction applied to the basilosaurids is not a universal correction for adnexa and cerebrospinal fluid, but is rather specific to the rete, a single component of the adnexa.

Ridgway et al. [2] recognized that adnexa (including the rete) of some extant cetaceans was both significant and variable in volume, and as a result questioned the validity of the existing brain mass estimates for fossil cetaceans. In the same paper Ridgway et al. [2] proposed that a regression of adnexa mass and body length in extant cetaceans could form the basis of an adnexa correction in fossil taxa. Boessenecker et al. [9] was the first to implement such a correction, and did so by regressing adnexa volume over a calculated endocranial volume in extant cetaceans using the brain and adnexa masses compiled by Ridgway et al. [2]. This regression equation was used to estimate the adnexa volume that would need to be subtracted from the endocranial volume of each fossil to approximate brain volume, and from this, a brain mass was calculated using the specific density of brain tissue [9] a method later followed by McCurry et al. [32].

Endocranial volumes have relevance to paleontologists as a proxy for brain size, but are generally not considered by anatomists who can simply weigh the brains of extant animals; as a result, endocranial volumes are rarely reported in anatomical studies. Using the limited data available, Boessenecker et al. [9] thoughtfully reasoned that reported adnexa and brain mass measurements reported in the literature could be by converted to volumes and used to recreate endocranial volume in extant taxa. Unfortunately, this approach does not take into account cerebrospinal fluid, another component to occupy the endocranial cavity in conjunction with the brain and adnexa. As we later demonstrate in extant taxa, leaving cerebrospinal fluid out of the reconstruction leads to underestimates of the true endocranial volume. In this study, we compile data to examine the relationship between endocranial volume and brain mass in placental mammals using only data in which the endocranial volume was measured directly, and to this we contribute new data on artiodactyls and cetaceans, and finally analyze this relationship to improve brain size estimates in fossil cetaceans while correcting using a Phylogenetic Generalized Least Squares (PGLS) regression.

### Estimation of body mass

Estimation of body mass in fossil taxa can be challenging and invariably estimates have some level of error [8, 52]. Despite some level of uncertainty in estimated body mass, body mass provides a useful measure with which many biological traits scale. Body masses of extinct mammals have been estimated using various skeletal metrics such as tooth size, cranial dimensions, length and diameter of long-bones, vertebral dimensions, and body length [8, 30, 53–60]. In fossil cetaceans specifically, body masses have been estimated using the width between the outer margins of the occipital condyles (OCW) [7, 9, 61, 62], serial regressions of vertebral dimensions [8, 30], and body length [8, 9, 14, 32, 47, 51]. Alternatively, body length has been estimated using multiple or linear regressions of cranial dimensions [52], or from postorbital width [63]. Such body length estimates may serve as intermediate proxies for body mass, or may be used in their own right as a measure of body size [52]. Most studies examining EQs in fossil cetaceans have used a combination of these techniques to estimate body mass. In general, body mass estimates for Eocene whales were derived from serial regressions of vertebral dimensions based on the work of Gingerich [8, 30], along with a few mass estimates based on body length [14, 32, 51]. In contrast, post-Eocene body mass estimates are derived almost exclusively from the OCW based estimates published by Marino et al. [7].

Gingerich [8] was critical of OCW derived body mass estimates citing what he considered the low R^2^ of 0.79 reported by Marino et al. [7] for their regression relating body mass to OCW. Boessenecker et al. [9] used the OCW body mass estimates of Marino et al. [7], but calculated alternative body masses for a few specimens using an equation of Pyenson and Sponberg [52] relating bizygomatic width to body length, in conjunction with a second equation [8] relating body length to body mass, a method followed by McCurry et al. [32] for fossil mysticetes. Boessenecker et al. [9] found that the body mass estimates generated using bizygomatic width were 136% larger compared to the OCW based mass estimates of Marino et al. [7], and concluded that additional studies on the estimation of body mass in fossil cetaceans were needed.

In this paper, we use occipital condyle widths (OCW) to predict body mass, which we find to have a high correlation with body mass in extant cetaceans using a phylogenetically corrected regression. OCW provides an attractive proxy for body mass because it is commonly preserved in fossils and can often be obtained in the same specimen from which the endocranial volume is measured. We propose that utilization of a single body mass estimation technique across the Eocene-Oligocene boundary will minimize methodological bias that could mimic temporal shifts in body mass and EQ through time.

### Encephalization quotient

The correlation between brain and body mass has long been recognized [64]. It was assumed that a given volume or mass of a mammal’s body requires a proportional volume or mass of brain tissue to fulfill generalized physiological functions such as thermoregulation and cell metabolism [5], and that deviations from this relationship represent increased or decreased capacity for “information-processing” [5, 6]:167). The encephalization quotient (EQ) [5], seeks to normalize the effects of brain and body mass scaling, producing a metric that is thought to better approximate the actual brain functioning capacity in a more meaningful manner than simple comparison of absolute brain masses between mammals. It should be noted that recently neurobiologists have begun looking at absolute numbers of neurons in the brain, rather than brain and body mass alone, as an increasingly important comparative tool in understanding cognitive ability [65]. In fossils, neuron number and density cannot be measured, but it is likely these methods will provide useful insights into interpreting relative brain size which can be obtained from fossils. EQ paints brain size evolution with a broad brush, and is not without complication, body mass for instance varies over the life of an individual, and thus its calculated EQ also changes. Although there are alternate methods that seek to observe allometries through time (e.g. Smaers et al. [31] and McCurry et al. [32]), EQ provides a baseline which is independent of the sample under study.

EQ is the ratio of the observed and predicted brain mass for a given body mass [5], as a result EQ is influenced by the reference sample from which the predicted brain mass is based. To determine the expected relationship between body and brain mass, a linear regression is executed on a dataset that traditionally includes mammals as a taxonomic class [5]. The implications of this regression’s slope have been much debated [5, 8, 28, 66], specifically whether the slope is closer to 0.66 or 0.75, which are thought to represent scaling factors inherent to mammalian physiological processes. Although the approach of using a reference population comprising all mammals is appropriate to study broad patterns of brain evolution, specific questions regarding a single group are better answered using regressions based on more taxonomically focused groups [11, 31, 67, 68], especially in cases where slopes of the regression for the study group deviate significantly from the broader sample that includes all mammals [11, 28, 31, 69]. Indeed McCurry et al. [32] proposed that constraints on maximum body size are relaxed for fully aquatic taxa and that this biases EQs calculations for fully aquatic cetaceans; although purely terrestrial artiodactyls are known to follow a different allometry than observed for an all mammal dataset [10]. Terrestrial artiodactyls include the land relatives of cetaceans [70–73], and we consider a regression based on this group, to provide a more appropriate baseline for examining the initial constraints on early cetacean brain evolution. In our view, this basis for calculating EQ best describes the scaling relation between body and brain size that shaped the origin of cetacean brain size evolution, although for comparison, we also present EQ values using the traditional all mammal scaling of 0.75.

## Materials and methods

### Ethics statement

No animals were killed for this study. Cetacean samples were obtained from animals harvested as part of the indigenous Iñupiat subsistence harvest, collected by the authors under the auspices of the Department of Wildlife Management, North Slope Borough. Terrestrial artiodactyl samples were obtained from local Ohio butcheries. Samples were brought onto the NEOMED campus with notification of IACUC consistent with NEOMED policy 3349-3-143. The authors assert that all procedures contributing to this work complied with the ethical standards, and that no study involved harming an endangered species.

### Institutional abbreviations

**AMNH**, American Museum of Natural History, New York, New York, USA; **BMNH**, British Museum of Natural History, London, UK; **CCNHM**, Mace Brown Museum of Natural History, Charleston, South Carolina, USA; **ChM**, Charleston Museum, Charleston, South Carolina, USA; **FMNH**, Field Museum of Natural History, Chicago, Illinois, USA; **GSP-UM**, Geological Survey of Pakistan—University of Michigan, Islamabad, Pakistan; **IITR-SB**, Indian Institute of Technology, Roorkee (previously RUSB); **NSB-DWM**, North Slope Borough, Department of Wildlife Management, Utqiaġvik, Alaska, USA; **SDSNH**, San Diego Natural History Museum, San Diego, California, USA; **UM**, University of Michigan, Ann Arbor, Michigan, USA; **USNM**, U. S. National Museum of Natural History, Washington, D.C., USA; **VPL**, Vertebrate Palaeontology Laboratory, Panjab University, Chandigarh, India; **YPM**, Peabody Museum of Natural History, Yale University, New Haven, Connecticut, USA.

### Brain mass and endocranial volume

A beluga head (NSB-DWM 2019LDL10) was provided by Iñupiat subsistence hunters in Point Lay, Alaska, in cooperation with the North Slope Borough, Department of Wildlife Management (NSB-DWM). Additional information on a bowhead whale (NSB-DWM 2008B11) expands on data published in Thewissen et al. [39] and Ridgway et al. [2], specifically providing data on adnexa mass. Pig and goat heads were received in fresh states from domestic animals culled for reasons unrelated to this research.

Brain masses were measured directly in un-fixed states on a laboratory balance after removal from the crania with the aid of hand tools and an oscillating bone saw. The pia and arachnoid mater are included in the reported brain masses as they are typically included in published brain masses. The pia and arachnoid, although nominally adnexa, are of negligible mass and impractical to remove without damaging the brain. In addition to brain mass, adnexa mass for one pig, beluga and bowhead were weighed (Table 1).

**Table 1:** Brain mass, endocranial volume and adnexa mass in terrestrial artiodactyls and cetaceans.

Specimen	Brain mass^1^ (g)	Endocranial volume^2^ (cc)	Adnexa mass^3^ (g)	Calculated brain volume ^4^ (cc)	Calculated % volume not occupied by brain^5^ (cm^3^)
Goat (A)	113.3	145	—	1089.4	24.6
Goat (C)	121.5	149	—	117.3	21.3
Goat (D)	102.1	130	—	98.6	24.2
Goat (E)	99.3	132	—	95.8	27.4
Pig (PGL 417)	128.5	186	—	124	33.3
Pig (PGL 419)	140	176	13.4	135.1	23.2
Beluga (NSB-DWM 2019LDL10)	2,074	2,528	165	1,994.2	21.1
Bowhead (NSB-DWM 2008B11)	2,948	8,400	1,238	2,834.6	66.3
Bowhead (NSB-DWM 2009B9)	2,980	8,900	—	2,865.4	67.8

^1^ Brain mass inclusive of pia and arachnoid.

^2^ Endocranial volume measured using beads or from CT-scan for the beluga.

^3^Adnexa mass including dura mater and rete weighed directly.

^4^ Brain volume calculated by dividing the brain mass by the density of neural tissue (1.036 g/cm^3^ for terrestrial artiodactyls, or 1.04 g/cm^3^ for cetaceans).

^5^ Calculated percent endocranial volume not occupied by brain.

Endocranial volumes were measured by occluding large foramina with clay and filling the cleaned cranial cavities with either 6 mm round plastic beads (or barley seeds) through the foramen magnum to the level of the occipital condyles (with the skull in a vertical position). Volumes were obtained by pouring the media from the cranial cavity into graduated cylinders. The beluga head was received and CT-scanned in a frozen state in a GE BrightSpeed 16 CT scanner housed at the at Metropolitan Veterinary Hospital in Akron, Ohio USA. The scan (Fig 1C and 1D) was digitally segmented to delineate the brain and cranial cavity volumes using the computer program Amira (Thermo Fisher). After scanning, the beluga head was thawed allowing extraction of the brain and adnexa. Damage done by the projectile that killed the animal was minimal, and the bullet was physically removed before recording the brain mass and was excluded from the segmented CT scan.

To estimate brain mass from endocranial volume in fossil taxa, we regressed brain mass over endocranial volume from data on extant mammals compiled from the literature, with the addition to new observations described here for beluga, pigs and goats (Table 1 and S1 Table in S2 File). We revisited the primary citations given in the compilations of Benoit [37] and Ridgway et al. [2] to ascertain whether the original authors measured endocranial volumes, or reported only brain and adnexa mass. From these we include only cases where endocranial volume was measured directly, as our new data (see Results and Discussion sections) indicate that brain and adnexa mass converted to volumes do not provide an adequate measure of endocranial volume. Following these criteria, we excluded the data for *Elephas maximus* and *Balaenoptera acutorostrata* in Benoit [37] because reported measurements in the original publications [75, 76] consisted of estimates of adnexa mass in the former, and neither included endocranial volumes. Of the data compiled in Ridgway et al. [2], we include only the specimen of *Tursiops truncatus* in which the brain volume was determined from a MRI scan [2], and presumably included an endocranial volume measurement from which Ridgway et al. [2] calculated percent adnexa.

From these data we derive an equation to predict brain mass from endocranial volume in a manner similar to Benoit [37], in which species averages for brain mass are regressed over endocranial volumes using a Phylogenetic Generalized Least Squares (PGLS) regression in R (version 3.5.1) [77] implemented using the R package Caper (version 1.0.1), with the phylogeny (S1 Data and S1 Fig in S1 File) from Upham et al. [78], and Caper set to estimate lambda using maximum likelihood. This final dataset of placental mammals included 26 extant species in 18 genera (S1 Table in S2 File) including species averages for our new data which consist of the pigs, goats and a beluga (Table 1).

### Estimating body mass from occipital condyle width (OCW)

To examine the relationship between occipital condyle width (OCW) and body mass in living cetaceans we compiled data from 112 extant cetacean individuals (pertaining to 35 species, of which 74 were adults) for which OCW and either body mass, or body length, were published (S2 Table in S2 File). Measuring body mass in large animals is logistically difficult. Some smaller cetaceans included in our study were weighed by the original authors as a whole, and some of the larger whales with published body masses were caught as part of commercial operations and were either weighed whole or in some cases in smaller segments. In the absence of reported body mass, body length was converted into an estimate of body mass using published equations relating body length to mass that were either species specific or genus specific (S2 Table in S2 File, notes). These equations are typically derived on actual measurements of body masses and lengths. As no published equations relating body mass and length for *Kogia*, *Mesoplodon*, *Hyperoodon* and *Indopacetus* were found, we compiled two separate datasets (one for *Kogia* and another for the three ziphiids) containing specimens of known length and mass and using a linear regression of log transformed data in Microsoft Excel derived equations for each group (S2 Table in S2 File). The species averages calculated by us are based on published records for individuals of those species, and not aggregated data on the species from reference works.

For the regression of body mass over OCW we limit the analysis to adults (n = 74), and calculate species averages for the 27 species in eight families, which are regressed using a Phylogenetic Generalized Least Squares (PGLS) analysis in R (version 3.5.1) [77] implemented using the R package Caper (version 1.0.1), using the phylogeny (S2 Data and S2 Fig in S1 File) of McGowen et al. [79], and Caper set to estimate lambda using maximum likelihood.

### Included specimens

Data for the Eocene archaeocetes (Table 2) including OCW and endocranial volumes were collected from the primary literature and are discussed individually in S1 Text. We concur with Gingerich [8] that the unusual vertebral geometry of *Basilosaurus* creates issues in estimating body mass. In addition, the published data available for the two species of *Basilosaurus* (*B*. *cetoides* and *B*. *isis*), which have entered the literature, are not reliable in our opinion and are excluded from our analysis (see S1 Text for further discussion).

**Table 2:** Eocene cetaceans included in this study.

	Species	Specimen number	Age (Ma)	Endocranial volume (cm^3^)	Endocranial volume reference	OCW (mm)	OCW reference
**Basilosauridae**							
	*Dorudon atrox*	UM 101222^endo^ ^OCW^	39	1173	Uhen [51]:p.362	126	Uhen [51]:p.574
	*Saghacetus osiris*	BMNH 10228^endo^ ^OCW^	39	480	Dart [33]:p.634	91.8	Kellogg [80]:p.246-247
	*Zygorhiza kochii*	USNM 16639^endo^ ^OCW^	37	917^**1**^	Marino et al. [14]:p.90,	112^**2**^	Kellogg [80] p.246-247
	*Zygorhiza kochii*	FMNH PM-459^endo OCW^	34	1189	Gingerich [81]:p.179	120	Gingerich [82]:p.168
**Protocetidae**							
	*Rodhocetus kasrani*	GSP-UM 3012^OCW^	47	290^3^	Gingerich [30]:p.434	88.7	Uhen, pers. com. (2020)
**Remingtonocetidae**							
	*Dalanistes ahmedi*	GSP-UM 3106^OCW^	45	400^3^	Gingerich [30]:p.434	104.8	Uhen, pers. com. (2020)
	*Remingtonocetus harudiensis*	IITR-SB 2770^endo OCW^	42	253	Bajpai et al. [83]:p.716	87	Bajpai et al. [83]:p.709

Ages are those of Boessenecker et al. [9] with the exception of *Remingtonocetus harudiensis* which comes from Bajpai et al. [83] and *Zygorhiza kochii* FMNH PM-459 which comes from Gingerich [82]. ^**endo**^, source of endocranial volume, ^**OCW**^, source of OCW, ^**1**^, endocranial volume was given for USNM 16638, possibly in error, see S1 Text for additional discussion, ^**2**^, OCW was reported for the *Zygorhiza kochii* “Millsaps College Museum (adult)” which we believe is now USNM 16639, see S1 Text for additional discussion, ^3^, indicates the value is not tracible to a numbered or vouchered specimen (see S1 Text).

For the post-Eocene fossil cetaceans we use data compiled in Boessenecker et al. [9], which is largely based on the dataset of Marino et al. [7], to this we add data for five fossil mysticetes from McCurry et al. [32]. Boessenecker et al. [9] in most cases used the body mass estimates of Marino et al. [7]. This dataset has reasonable temporal coverage of Neogene cetaceans, although certain taxa are overrepresented (eurhinodelphinids). Such sampling biases can affect conclusions about patterns of evolution. Both publications [7, 9] include body mass estimates, although the OCW values from which the body masses are derived are not included in either publication. We obtained OCW measurements from either published sources or from measurements generously provided by Mark D. Uhen (personal communication, 2020), a co-author of Marino et al. [7]. For specimens for which we could not obtain OCW values, we used the regression equation of Marino et al. [7], solved for OCW to obtain the original OCW values from the published body masses. To our knowledge, Marino et al. [7] did not publish the equation, although Boessenecker et al. [9] later published it with an attribution to Marino et al. [7]. Comparison of OCW values obtained from either Mark Uhen or the literature, with those of the back-calculated values, show them to be nearly identical providing confirmation that our back-calculated values are suitable to use in cases in which OCW measurements could not be obtained. We include five fossil mysticetes recently published by McCurry et al. [32] and for these we estimate brain mass from their published values and obtain the occipital condyle widths to estimate body mass from the literature, and in one case from a CT scan and an estimate from a published figure (See S3 Table in S2 File). Ridgway et al. [2] compiled brain and body masses for extant cetaceans, and from this publication we calculated species means for the brain and body masses of the adult specimens (n = 45).

Fossil and extant terrestrial artiodactyl data come from Orliac and Gilissen [35]. For the fossil artiodactyls in that dataset (n = 35), we follow the reported body mass estimates, and use our new equation (see Results section) to estimate brain mass from the endocranial volumes published by Orliac and Gilissen [35]. The brain and body masses for the extant artiodactyls (n = 73) compiled in Orliac and Gilissen [35] are included as published.

### Calculation of encephalization quotients

We calculate EQ as the ratio of observed brain mass over the expected brain mass for an animal of the same mass [5]. Based on published body and brain mass scaling coefficients in Burger et al. [11] we calculate two sets of EQ values, one, EQ_0.56_ using the scaling coefficient observed in extant terrestrial artiodactyls (Eq 1), and a second, EQ_0.75_ based on all mammals (Eq 2).


$${EQ}_{0.56}=\frac{brain\;mass}{10^{\left(0.56\times \log_{10}\left(body\;mass\right)-0.44\right)}}$$ Eq 1

$${EQ}_{0.75}=\frac{brain\;mass}{10^{\left(0.75\times \log_{10}body\;mass-01.26\right)}}$$ Eq 2

To test the difference between the species means for the middle Eocene archaeocetes (n = 3, remingtonocetids and protocetids), late Eocene basilosaurids (n = 3 species, 4 individuals), and the Oligocene (n = 4) odontocetes, we performed an ANOVA, to test significance, followed by Tukey’s Honest Significant Difference (HSD) post-hoc test using the base functions in R (version 3.5.1) [77]. Data for this test is comprised of the species mean log_10_ EQ_0.56_ scores of all Eocene archaeocetes and Oligocene odontocetes.

## Results

### Brain mass and endocast volume

#### New data on cetaceans and terrestrial artiodactyls

Measurements of endocranial volume, adnexa mass, and brain mass of beluga, bowhead, goats, and pigs are presented in Table 1. These data, in addition to previously published values (S1 Table in S2 File) are used to examine the relationship between brain mass and endocranial volume in extant taxa, the quantification of which forms the basis of our brain mass estimates in fossil cetaceans and terrestrial artiodactyls. The specimens for which we record adnexa mass are used to illustrate that adnexa and brain mass, converted to units of volume, do not closely approximate the actual endocranial volume.

**Fig 2:**
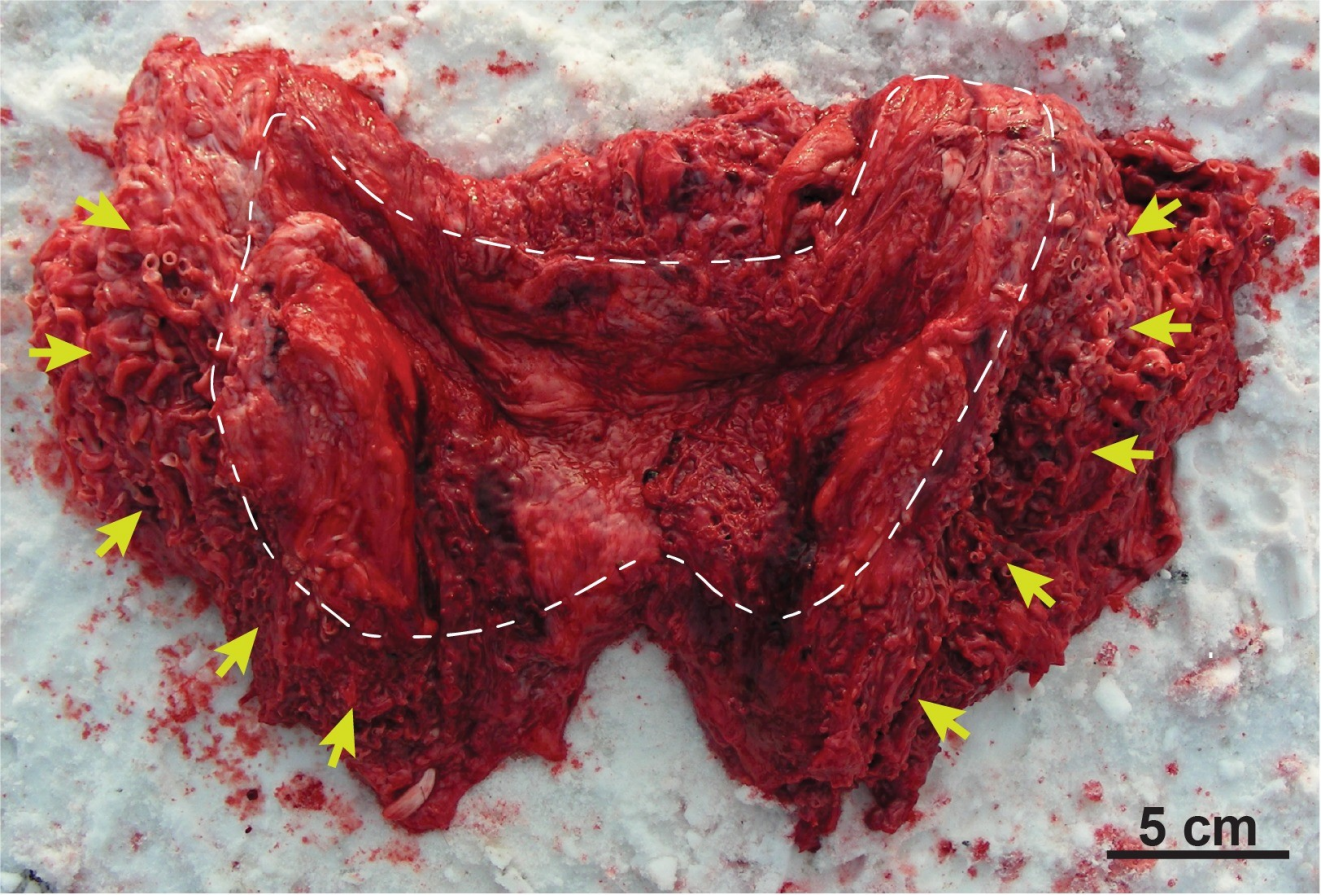
Bowhead rete. Excised section of a bowhead whale (NSB-DWM 2008B11) rete; this portion of the rete is located on either side of the cerebellum; the smooth part of the dura touches the arachnoid (inside white dashed line); yellow arrows indicate the reticulate network of blood vessels that compose the rete.

Thewissen et al. [39] and Ridgway et al. [2] published data on bowhead whale brain mass; here we supplement those accounts with additional data on adnexa mass. Bowhead whale NSB-DWM 2008B11 had a brain mass of 2,948 g [39], an adnexa mass (including the rete mirabile, Fig 2) of 1,238 g, and an endocranial volume of approximately 8,400 cm^3^ [39]. Using these data, we can estimate the percent endocranial cavity not occupied by the brain. To estimate brain volume, the brain mass is divided by the specific density of cetacean brain tissue, 1.04 g/cm^3^ [2], resulting in a volume of 2835 cm^3^. Subtracting the brain volume from the endocranial volume indicates that 5565 cm^3^, or 66%, of the cranial cavity is filled with adnexa and cerebrospinal fluid. The availability of an endocranial volume for this bowhead is unique in that this information is not typically reported for extant taxa. The limited availability of paired brain mass and endocranial volumes has led researchers wanting to estimate brain mass in fossil taxa, or even in extant specimens, to use adnexa and brain masses to reconstruct endocranial volume. The data we describe herein includes brain and adnexa mass, in addition to endocranial volume which allow us to illustrate issues that arise when endocranial volumes are not recorded.

**Fig 3:**
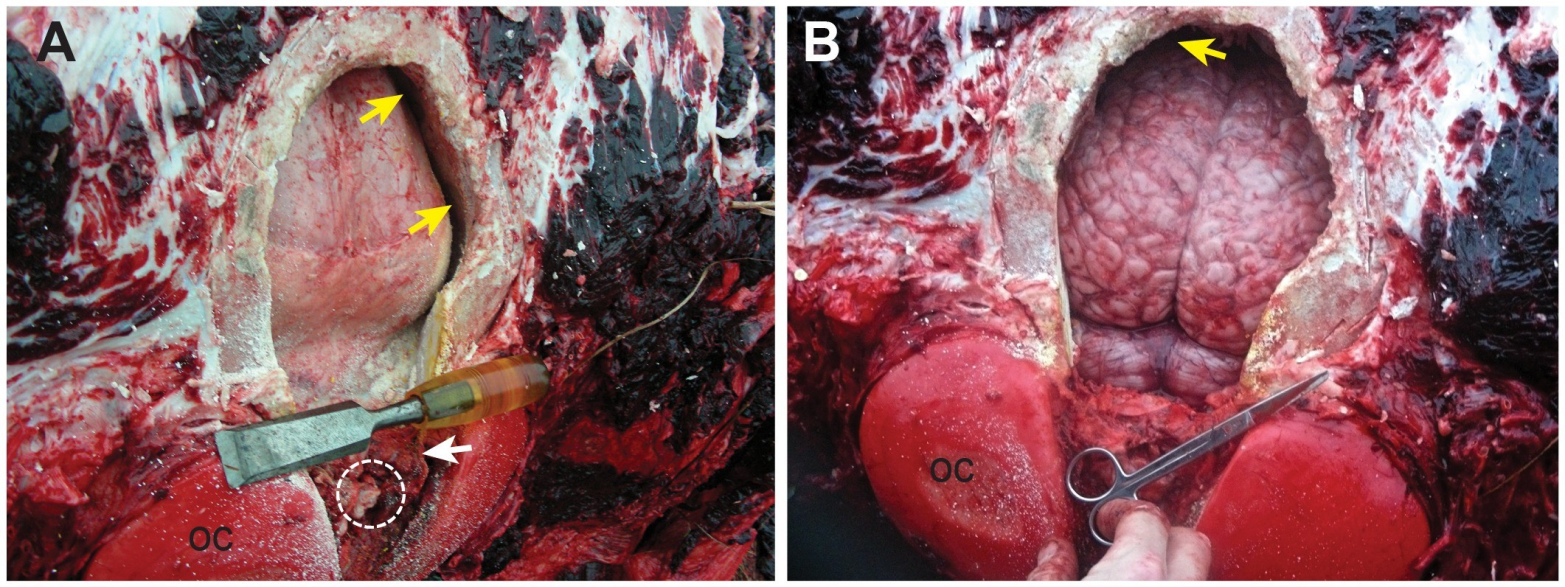
In situ bowhead whale brain. Bowhead (NSB-DWM 2009B11) with the calvarium removed exposing the brain; yellow arrows indicate now empty space between the brain and calvarium; oc, occipital condyle; (A) brain covered by intact dura which has collapsed onto the brain after the cerebrospinal fluid leaked out; note the small size of the spinal cord (white dashed circle) which is surrounded by the cranial rete mirabile (white arrow). (B) brain with dura removed exposing gyri of the cerebrum (upper) and cerebellum and brainstem (lower).

As an example, if the measured endocranial volume of the bowhead was not recorded, using the combined brain and adnexa mass (converted to volume assuming a neural-tissue density of 1.04 g/cm^3^) would suggest a theoretical endocranial volume of 4,025 cm^3^. In this case, only 30% of the cavity (as compared to 66% when calculated using the measured endocranial volume) would be assumed to contain adnexa and cerebrospinal fluid. The difference between these values is a result of the cerebrospinal fluid volume, which is not reflected in the estimate for which endocranial volume was reconstructed. Observations made during extraction of a bowhead brain help illustrate the magnitude of lost cerebrospinal fluid volume which can be visualized by the large gap between the braincase and dura (Fig 3).

Results for the beluga (NSB-DWM 2019LDL10) further illustrate that measurements of endocranial volume cannot be accurately reconstructed from brain and adnexa mass. Segmentation of the CT scan shows the brain fills 79% of the cranial cavity (Table 1) leaving 21% of the cavity to contain adnexa and cerebrospinal fluid. In contrast, if the endocranial volume is reconstructed from brain and adnexa mass, the brain would be assumed to fill 93% of the cranial cavity.

For the one pig in which adnexa mass was measured, the brain filled nearly 77% of the cavity leaving 23% filled with adnexa and cerebrospinal fluid. However, when the endocranial volume measurement is reconstructed, the adnexa and cerebrospinal fluid would be erroneously assumed to represent only 9% of the volume. The difference is again attributable to cerebrospinal fluid loss.

The four goat samples indicate that while brain masses vary by around 20% intraspecifically, endocranial volumes vary by more than 30% (Table 1). The brain fills on average 75% of the cranial cavity (Table 1) leaving 25% for adnexa and cerebrospinal fluid.

#### Brain mass and endocranial volume allometry

**Fig 4:**
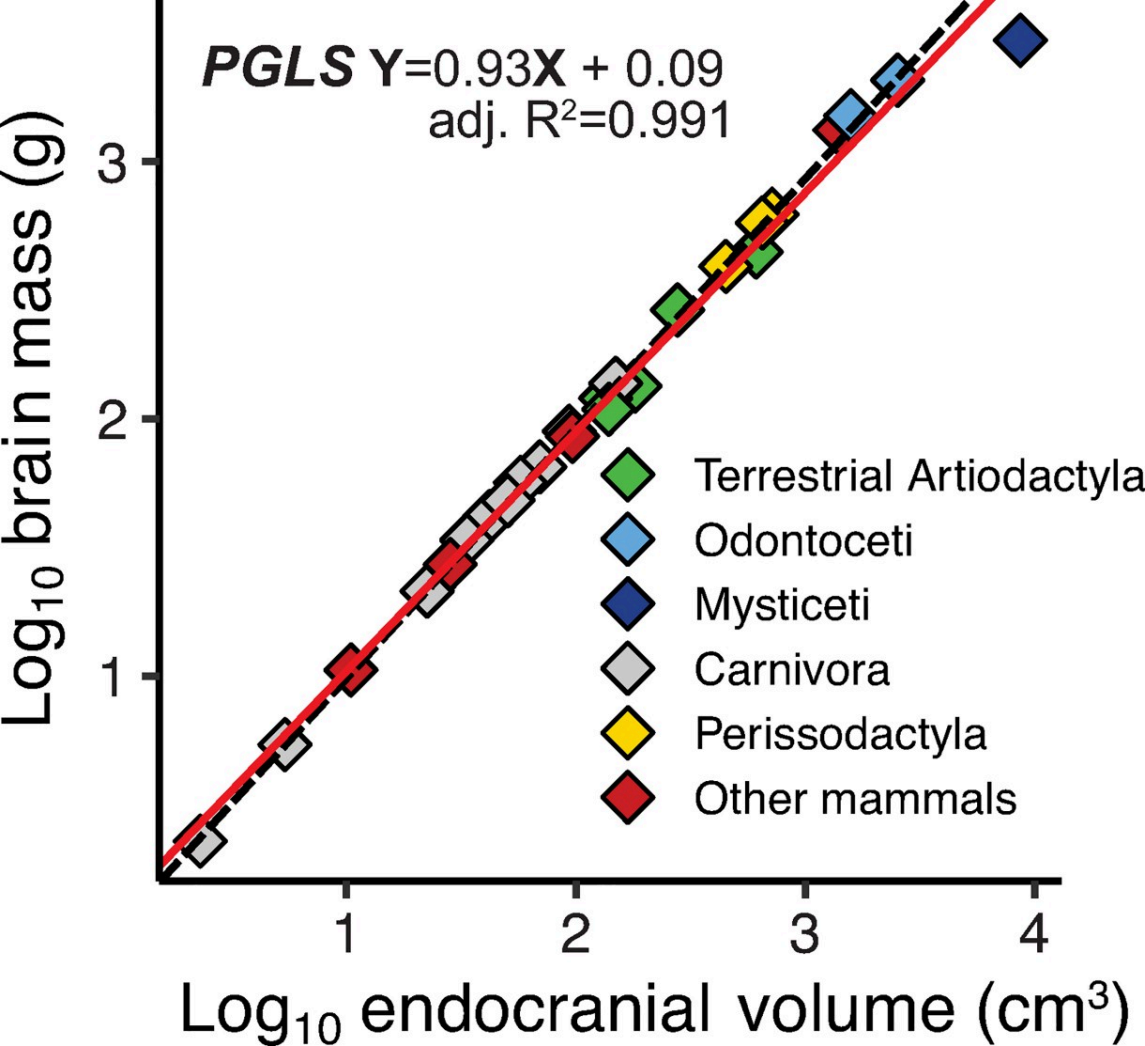
Plot of log transformed brain mass and endocranial volumes. Scaling of brain mass and endocranial volume in adult extant placental mammals (species averages); the bowhead (dark blue diamond, farthest upper-right) is the only mysticete.

To establish the allometric relationship between endocranial volume and brain mass in extant mammals we examine published values supplemented with new data for pigs, goats, and a beluga and bowhead whale (Fig 4, Table 1 and S1 Table in S2 File). Results of the PGLS regression of the log-transformed brain mass (grams) and endocranial volume (cm^3^) give Eq 3

$$Log_{10}(brain\;mass)=0.933\;Log_{10}(endocranial\;volume)+0.088$$ Eq 3

This regression is based on species means of 611 specimens (which includes 26 species within 18 genera) results in an adjusted R^2^ of 0.991, and a standard error of ± 0.018 for the slope, and ± 0.04 for the intercept, with a lambda of 1X10^-6^.

This equation (Eq 3) is used to estimate brain mass from endocranial volume in the fossil artiodactyls compiled by Orliac and Gilissen [35], the post-Eocene fossil cetaceans described by Marino et al. [7] and Boessenecker et al. [9], the Eocene archaeocetes (Table 2 and S3 Table in S2 File), and fossil mysticetes described by McCurry et al. [32]. Results for the Eocene archaeocetes, including brain mass estimates from previous studies, are given in Table 3, and data for the post-Eocene fossil cetaceans in S3 Table in S2 File.

**Table 3:** Eocene archaeocete body and brain mass estimates.

	Body mass estimates (kg)					Brain mass estimates (g)				
Taxon	Gingerich 1998	Marino et al. 2004	Gingerich 2016	Boessenecker et al. 2017	This study	Gingerich 1998	Marino et al. 2004	Gingerich 2016	Boessenecker et al. 2017	This study
*Dorudon atrox*	1,140^V^	2,240^L^	1,126	2,240^V^	**1023** ^C^	960	1185.4^3^	944	931.4	**883**
*Saghacetus osiris*	350^V^	350 ^V^	350	350^V^	**379** ^C^	388	388	388	373	**383**
*Zygorhiza kochii* ^1^	—	2040^L^	—	998^V^	**707** ^C^	—	800.8	—	702.3	**701**
*Zygorhiza kochii* ^2^	—	—	998	998^V^	**877** ^C^	—	—	960	920.2	**894**
*Rodhocetus kasrani*	590^V^	290*	590^V^	290*	**340** ^C^	290	291	291	272.7	**240**
*Dalanistes ahmedi*	750^V^	750^V^	750^V^	750^V^	**574** ^C^	400	400	400	372.2	**323**
*Remingtonocetus harudiensis*	—	—	—	—	**320** ^C^	—	—	—	—	**211**

^C^, body mass from OCW.

^V^, body mass based on vertebral dimensions

^L^, body mass from body length

*, uncertain origin

^1^, USNM 16639

^2^, FMNH PM-459

^3^, 20% rete correction apparently not applied.

See S1 Text for full explanations of previously published body and brain mass estimates.

### Occipital condyle width and body mass

**Fig 5:**
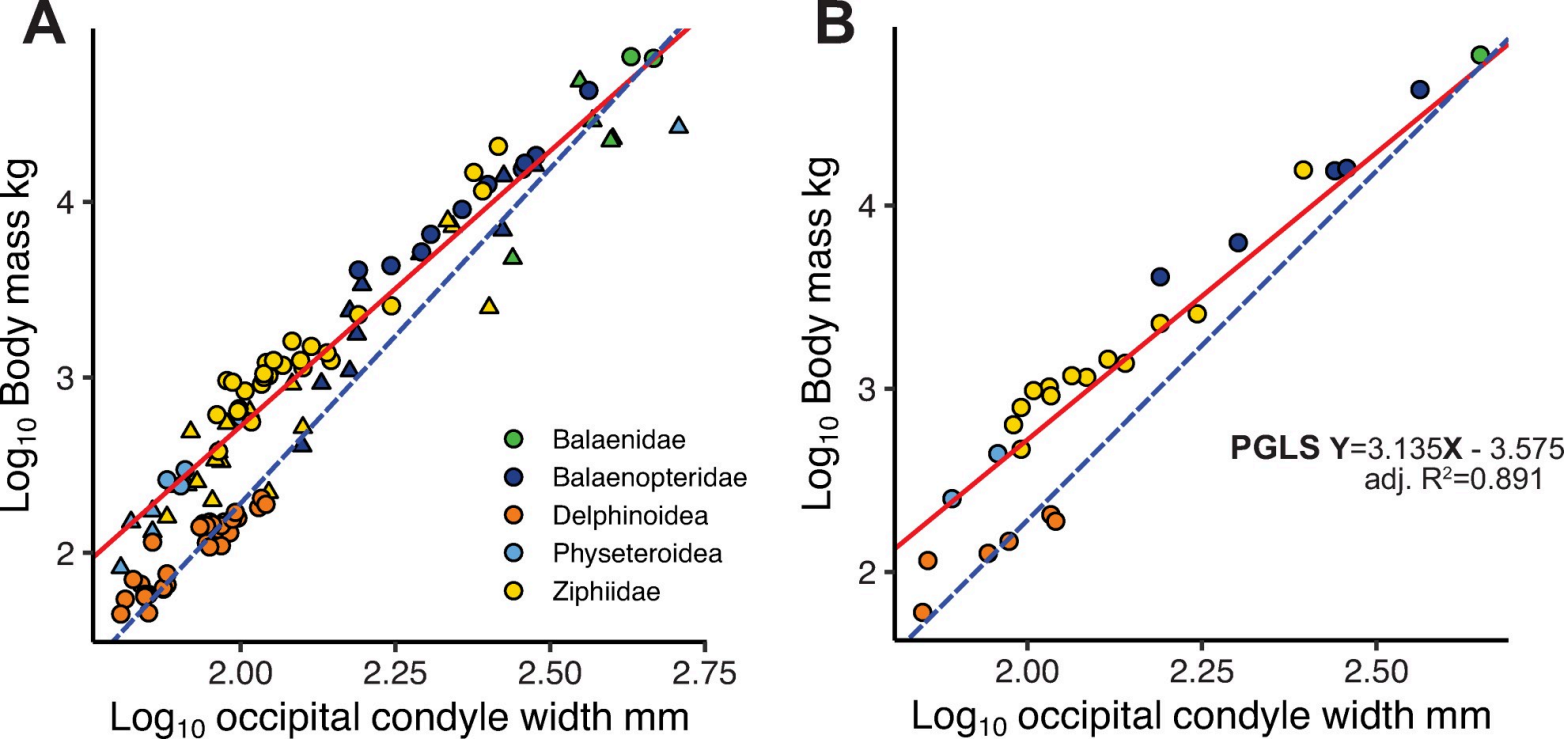
Scaling of occipital condyle width (OCW) and body mass in extant cetaceans. Red line shows our PGLS regression (Eq 3) for adults (species averages) in both plots. (A) Plot including all data (S2 Table in S2 File); circles represent adult individuals, triangles represent sub-adults; blue line represents the regression of Marino et al. [7] reported in Boessenecker et al. [9]. (B) Plot of adult species means which form the basis of our regression (red line).

We establish the relationship between occipital condyle width (OCW) and body mass in extant cetaceans to estimate body mass in fossil cetaceans. Initial examination of the data (Fig 5A), show the delphinoid taxa (for which we have data) to diverge from the remaining taxa. The regression of Marino et al. [7] (published in Boessenecker et al. [9]) is plotted in Fig 5A and can be seen to pass through the delphinoids. Given the data available to us, it would appear the regression of Marino et al. [7] was heavily influenced by delphinoids, although this cannot be verified, as the calibration dataset on which Marino et al. [7] based their equation is not available.

The fossil cetacean dataset compiled by Marino et al. [7] and Boessenecker et al. [9] includes only five delphinoids, and for those we retain the body masses originally published in Marino et al. [7] as our data would suggest their estimates are likely more appropriate (Fig 5A). The final PGLS regression (Eq 4) that describes the relationship between OCW and body mass is based on adult species means consisting of 74 specimens, representing 27 species within eight families.


$$Log_{10}(body\;mass)=3.135\;X\;Log_{10}(OCW)–3.575$$ Eq 4

Where body mass is in kilograms, and occipital condyle width (OCW) in millimeters; the equation produces an adjusted R^2^ = 0.87, and a standard error of ± 0.232 for the slope, and ± 0.52 for the intercept, with a lambda of 0.942.

### Fossil cetacean and terrestrial artiodactyl EQs

**Fig 6:**
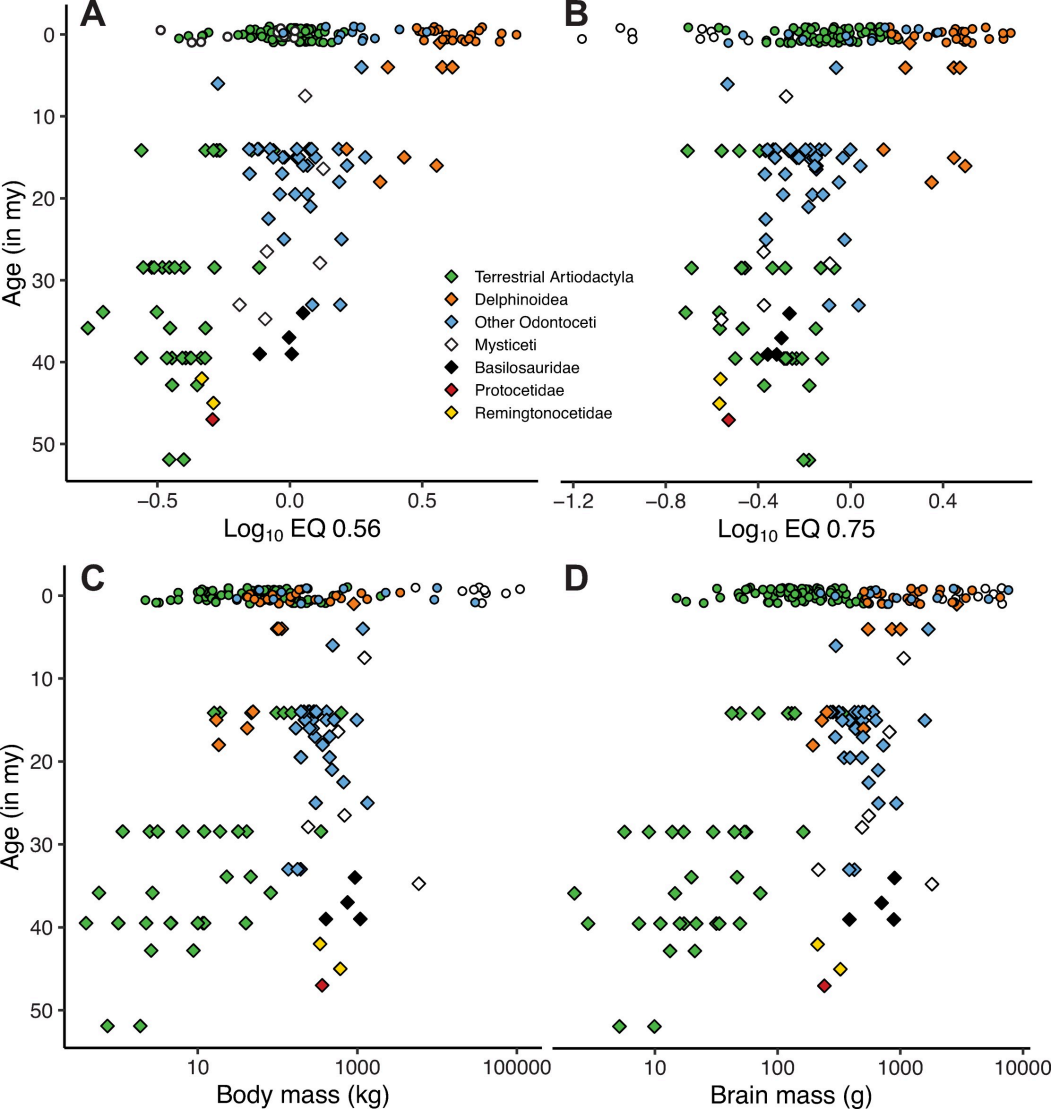
EQ_.56_, EQ_.75_, and body and brain mass through time for cetaceans and terrestrial artiodactyls. Age in millions of years before present; extant specimens (circles) are randomly dispersed around the zero datum to increase visibility.

EQ_0.56_, EQ_0.75_, and body and brain mass for terrestrial artiodactyls and cetaceans through time are given in Fig 6.

### Eocene cetacean EQs

In addition to the methodological improvements in estimating values needed to calculate EQs, we make some preliminary observations in regarding the Eocene archaeocetes. In particular, we examine whether there are differences between middle Eocene archaeocetes (remingtonocetids and protocetids), late Eocene basilosaurids, and Oligocene odontocetes. S4 Table in S2 File presents the results of a Tukey’s Honest Significant Difference test and shows significant differences between middle Eocene and late Eocene archaeocetes (p < .001), but not between late Eocene basilosaurids and Oligocene odontocetes (p>.1).

## Discussion

In this paper we refine the brain and body mass estimates commonly incorporated in studies of fossil cetacean brain size evolution [7, 9, 12, 15, 21, 23, 24, 30–32]. We reassess the methods used to estimate brain mass in both fossil cetaceans and their terrestrial artiodactyl relatives and reexamine and improve the methods used to estimate body size in fossil cetaceans. We evaluate the choice of reference group used to calculate EQ, and correct errors that have continued to propagate in the literature. These improved brain and body mass estimates are then incorporated into revised EQs (Fig 6).

### Brain mass estimates

The allometry between brain mass and endocranial volume in modern taxa forms the basis for estimation of brain mass in fossils. The cranial cavity, along with the brain, contains cerebrospinal fluid, and the remaining tissues, collectively termed adnexa. The adnexa and cerebrospinal fluid are both a volumetrically significant and variable occupant of the cranial cavity. As such, the endocranial volume of fossils should not be equated with brain volume (or mass) without taking the adnexa and cerebrospinal fluid into account. We further demonstrate that in compiling data to establish the allometry between brain mass and endocranial volume in extant taxa, adnexa and brain mass alone cannot be used to reconstruct endocranial volume (in cases where it was not originally measured), as this ignores the contribution of cerebrospinal fluid volume and results in an underestimate of the endocranial volume.

Jerison [5] recognized that cetacean brains do not fill the entire cranial cavity, although he assumed the difference between brain and endocranial volume was small enough to not influence the observation of large-scale trends. In contrast, the variability of adnexa size exhibited in modern cetaceans, which partially accounts for the difference between endocranial and brain volume, led Ridgway et al. [2] to question the validity of existing fossil whale brain mass estimates. Addressing this problem, Boessenecker et al. [9] used published brain and adnexa masses in extant cetaceans as a basis for improving brain mass estimates by predicting the adnexa volume that needed to be subtracted from the endocranial volume to approximate brain volume (and mass) and this approach was followed by McCurry et al. [32].

The approach of Boessenecker et al. [9] was an improvement, especially considering an adnexa volume correction had never been factored into the original brain mass estimates of Marino et al. [7] (with the exception of the basilosaurids, discussed below). We refine the approach of Boessenecker et al. [9] with additional data to better assess the allometry of brain mass and endocranial volume in extant cetaceans and terrestrial artiodactyls (Fig 4 and Table 1). Given the limited data, our regression includes individuals from many placental orders (S1 Table in S2 File). New observations (Table 1) show that cerebrospinal fluid is a volumetrically significant component of the cranial cavity and demonstrates that brain masses of fossil taxa will be overestimated if the combined brain and adnexa mass is converted to volume and equated with endocranial volume. To remedy this, we include only specimens in our calibration dataset of extant taxa for which both endocranial volume and brain mass are available (Fig 4). The resultant correlation (Eq 3) between endocranial volume and brain mass is remarkably robust within the taxa examined with an adjusted R^2^ = 0.991, based on a PGLS regression, and the lambda value close to zero indicates the absence of a strong phylogenetic signal. Our results show the brain fills (by volume) approximately 33 to 98 percent (or 70–98 percent if the only mysticete for which there is data is excluded) of the cranial cavity in extant placental mammals (including terrestrial artiodactyls and cetaceans), with the percentage decreasing with increasing body size. Within the body size range of the fossil cetaceans examined (14–1336 kg) the brain is predicted to have occupied between 66–81 percent of the cranial cavity.

Brain mass and endocranial volume are only available for two extant mysticete bowhead whales (Table 1), the oldest individual is plotted in Fig 4. Although we include the bowhead in the regression, the available data has limits and is based on a single individual. First, the data are not from a fully-grown individual. Second, this mysticete is significantly larger than the fossil taxa for which we estimate brain mass and its extreme size could unduly influence the regression. Third, mysticete whales are commonly identified as having an enlarged cranial rete in comparison with odontocetes and terrestrial artiodactyls [2, 45, 46, 83, 84].

The outlier status of the bowhead, in terms of scaling, suggests mysticetes could be exceptions to the general allometry between brain mass and endocranial volume (Eq 3 and Fig 4) to which the smaller terrestrial artiodactyls appear to follow. However, it is also possible that bowheads are simply following the expected allometry of terrestrial artiodactyls, as our currently limited data might not resolve that relationship as the larger artiodactyls remain unsampled. Without additional data, we cannot determine if an additional or second correction is needed to estimate brain mass from endocranial volume in fossil mysticetes, and if that correction would pertain to all artiodactyls that possess a rete. Without a clearer understanding of brain mass and endocranial volume allometry, brain mass and EQ estimates for fossil mysticetes should be treated with caution.

Most authors have assumed that fossil basilosaurid cetaceans, like the extant mysticetes, possessed a relatively enlarged cranial rete. Gingerich [30] subtracted 20% from the endocranial volume of some basilosaurids to estimate brain mass, based on work of Uhen [51], and Marino et al. [7, 14] extended this correction to the remaining basilosaurids. Our data indicate that endocranial volumes of terrestrial artiodactyls and cetaceans would need a volumetric reduction of approximately 24 percent (the average for the fossil whales in our dataset) to approximate brain volume directly from endocranial volume, and this correction is without the assumption that an enlarged rete is present.

In the case of the basilosaurids, even if they possess an enlarged rete, it remains possible they follow the mysticete scaling pattern (if the mysticetes have a unique allometry). Without the data needed to account for scaling of an expanded rete, if needed, to make a more sophisticated correction, for rete scaling in basilosaurids and mysticetes, and with the acknowledgement that it is likely that the correction is not a fixed percentage, we believe that the evolutionary simpler explanation is that basilosaurids follow the same rete scaling rules as terrestrial artiodactyls and other mammals withing the body size range examined (Table 3).

### Body mass estimates

In studies of brain evolution, Eocene cetacean bodyweights have been estimated using vertebral dimensions and body length, whereas weights for post-Eocene cetaceans have been almost exclusively based on occipital condyle width [7, 9, 30]. As a result, these studies have found the Eocene-Oligocene boundary coincided with an apparently rapid shift in EQ. Our data suggest that this apparent shift is an artifact caused by the abrupt change in the method used to estimate body mass, and that it does not represent an actual shift in EQ (Fig 6 and S3 Table in S2 File). Using a single bodyweight estimation method across the Eocene–Oligocene boundary provides the methodological consistency needed to reduce the appearance of artifacts.

Selection pressures for increased or decreased body sizes can have a significant effect on EQ trends (e.g. Smaers et al. [31] and Montgomery et al. [12]) and, on a more basic level, body size estimates have a profound effect on the calculation of EQ values. It is therefore critical to carefully evaluate body size estimates of fossil taxa as they are important in assessing relative brain size. With the great diversities of body shapes expressed across terrestrial artiodactyls, Eocene cetaceans, and post-Eocene cetaceans, a skeletal proxy with a single calibration dataset cannot be expected to provide reasonable body mass estimates in all groups and time periods. Our study shows that in living adult cetaceans, body weights correlate well with OCW (adjusted R^2^ of 0.89). Although the few delphinoid taxa for which we have data appear to follow alternate scaling coefficients and if they are excluded from the regression, the adjusted R^2^ increases to 0.98. Estimating body size based on several skeletal proxies using a multiple regression approach has clear advantages [52], although variability in the preservation of fossils limits the number of specimens for which sufficient data can be obtained as fossils are rarely preserved or collected as complete skeletons.

Our bodyweight estimates for Eocene archaeocete cetaceans (Table 3) broadly agree with those of Gingerich [8, 30] whose estimates are based on vertebral dimensions. In contrast, our estimates differ from those of Marino et al. [7] for the Eocene and post-Eocene cetaceans (Table 3 and especially S3 Table in S2 File). For the post-Eocene cetaceans, our estimates, and those of Marino et al. [7], share the use of OCW as a skeletal proxy, but are based on different calibration datasets. The divergence between our estimates and those of Marino et al. [7] is therefore not driven by the choice of skeletal proxy, but rather by the data used to establish the relationship between OCW and body mass in extant cetaceans.

In scrutinizing the relationship between OCW and body mass in modern cetaceans it becomes obvious that delphinoids (Fig 5A) follow a divergent allometry from other cetaceans. Gingerich [8] rejected the use of OCW to estimate bodyweight based on what he considered a weak correlation by Marino et al. [7] who reported a R^2^ of 0.79. Our work shows that this concern is now alleviated (R^2^ of 0.89 for our PGLS regression). A plot of our raw data illustrates why the alternate OCW and body mass scaling of the delphinoids may not have been immediately apparent if the immature individuals (Fig 5A, triangles) are included as they partially mask the distinct delphinoid scaling. When the OCW to body mass regression of Marino et al. [7] as given in Boessenecker et al. [9] is plotted with our data (Fig 5), it would appear to have been driven by delphinoid taxa (especially at the lower end of the observed body masses) suggesting the calibration dataset of Marino et al. [7] contained a significant delphinoid component. Conversely, this indicates that, for the delphinoids, Marino’s regression likely provides more accurate body size estimates, and we retain the body masses of Marino et al. [7] for this group in our analysis. As we have a rather limited sampling of delphinoid species as this time, we do not generate a separate regression in this study, and would note that it remains unclear if all delphinoids follow this pattern.

The high correlation between OCW and body mass in our sample instills confidence in this method of bodyweight estimation. Using OCW to estimate bodyweight has the additional advantage that a single skull can be used to estimate both brain and body mass. Rarely do fossils consist of complete skeletons, as a result bodyweight estimates made using vertebral columns or body length are often based on composites of multiple incomplete specimens. Thus, endocranial volume and skull measurements may be derived from different individuals and such use of multiple individuals has the potential to introduce artifacts arising from intraspecific variation. Standard deviations of bodyweight in cetaceans within the same population are around 10% of the mean (e.g., *Phocoena* [85]), implying the introduction of noise when combining measurements of several individuals. In the case of fossils, time-averaging introduces a temporal component, such that the specimens are unlikely to have come from even a single population. However, limiting a study to include only data based on a single specimen lowers the overall sample size, and for the middle Eocene whales, this restriction would reduce the already small sample size (Table 3 and S1 Text). We chose to include these few composite specimens in spite of the drawbacks, but remain mindful that some weaknesses may exist.

While the use of OCW to estimate body size has advantages, we do not argue for its indiscriminate use. We utilize OCW to estimate bodyweights in fossil cetaceans, but for the fossil terrestrial artiodactyls we use the published body masses of Orliac and Gilissen [35] which are based on dental dimensions rooted on regressions of taxonomically specific artiodactyl subclades. Although OCW has been used to estimate body mass in fossil terrestrial artiodactyls [54, 56, 86], the paired OCW and body mass data we have compiled to date is based on fully aquatic forms and would likely be unsuitable for more terrestrial forms. The needed introduction of older, more transitional, fossil cetaceans (such as pakicetids and ambulocetids) into future analyses will provide additional challenges in balancing the use of more taxon specific body mass estimation methods, with the dangers of introducing potential artifacts as we described at the Eocene-Oligocene boundary.

## Conclusions

The rete mirabile, one component of the adnexa, is present in most Cetartiodactyla, and is extensively developed in mysticetes. This may be illustrated by the observation of Duffield et al. [87] that the bowhead whale brain can be extracted without damage through the foramen magnum if the rete which surrounds the spinal cord and occludes this opening is removed. Data specifically on rete size in mysticetes is sparce, and paired brain mass and endocranial volumes for physically mature mysticete specimens are unavailable. Terrestrial artiodactyls and odontocetes also possess an endocranial rete mirabile, and although the interspecific scaling between brain mass and endocranial volume remains relatively understudied, there is significantly less data pertaining to specific to scaling of the rete. Thus, it remains possible, but untested, that the rete, as a component of the adnexa in mysticetes, does in fact scale with endocranial volume or brain mass, in a manner similar to terrestrial artiodactyls and odontocetes. In this case the apparent disproportionally large rete observed in mysticetes is simply the expected product of the enormous body sizes they attain compared to odontocetes and terrestrial artiodactyls.

The unusual morphology of basilosaurid endocasts [33, 48] has been interpreted by most authors as evidence they possessed an unusually large rete mirabile, and most modern researchers have followed Uhen [51], Gingerich [30] and Marino et al. [14] in assuming that a rete filled a fixed 20 percent of the basilosaurid cranial cavity. Our data, which does not specifically address the rete in isolation from the adnexa, shows that the percentage of the cranial cavity filled by adnexa and cerebrospinal fluid is not fixed, but rather scales with endocranial volume. This implies that a correction in the form of a single fixed percentage may not fully capture adnexa volume.

It remains unclear if mysticetes share the same scaling with all artiodactyls. If mysticetes do follow an alternate scaling relationship, the possibility exists that basilosaurids would scale in a similar manner, conversely, they may follow the general scaling which we demonstrate and includes the terrestrial artiodactyls we have examined. Further study of mysticetes and large bodied terrestrial artiodactyls may lead to a better understanding of the relationship between brain mass and endocranial size in taxa that appear to possess a disproportionately large rete. A more complete understanding of this relationship will help improve brain mass predictions in both fossil mysticetes and basilosaurids.

We take the coincidence of our body mass estimates using OCW with those of Gingerich [8], which are based on vertebral column measurements, as general validation of both methodologies. As more complete fossil specimens become available, a method that uses multiple skeletal proxies, such as that of Pyenson and Sponberg [52] may provide further fidelity to the estimates, but would potentially limit the number of included specimens.

We suggest the reference sample used as the basis to calculate EQ should be taxonomically constrained when the focus is on the evolution of a specific group. There is a rich literature discussing the reasons that empirical scaling coefficients gravitate toward specific values (see summaries by Martin et al. [69], Armstrong [88], Boddy et al. [10] Smaers et al. [31]). A slope closer to 0.67 is thought to reflect the relation between brain mass and body surface area [64, 89, 90]. A slope closer to 0.75 [10, 69] is commonly assumed to reflect a link with metabolic rates [66, 69]. However, neurobiologists have pointed out that constraints on brain size in mammals across the seven orders of magnitude in body weight is more likely related to constraints within the brain [91]. That would imply that a single, generalized mammalian scaling coefficient does not clarify our understanding of the evolution of specific taxonomically limited groups.

Fig 6 presents data that bear on multiple aspects of brain and body size evolution in terrestrial artiodactyls and cetaceans. Our focus relates to relative brain size in Eocene archaeocete, and its possible relation to the large brains in odontocetes. Our observations are currently based on just three species of middle Eocene fossil cetaceans (two remingtonocetids, one protocetid) and three basilosaurids (four specimens). We investigated mean EQ values in middle Eocene archaeocetes (protocetids and remingtonocetids), late Eocene basilosaurids, and Oligocene odontocetes (S4 Table in S2 File). Middle Eocene cetaceans have statistically different EQs from late Eocene cetaceans. This finding is at odds with most studies [3, 9], but consistent with the findings of Gingerich [8] and Montgomery et al. [12].

Most authors have identified the Eocene-Oligocene boundary as a time in which cetaceans show a sudden increase in EQ [7–9, 92]. However, our analysis does not support this. The increase observed in previous studies is, at least in part, an artifact caused by differences in body mass estimation methods employed across that interval. Our post-Eocene body mass estimates are significantly greater than those of Marino et al. [7] and Boessenecker et al. [9] (S3 Table in S2 File), while our Eocene body mass estimates are similar to those of Gingerich [8]. This presumed rapid EQ increase was interpreted as evidence that large relative brain sizes in odontocetes evolved around the same time as echolocation [18, 19], or as adaptations to cooling climates [23, 24]. Our results suggest that the evolutionary pattern cannot support these hypotheses in its present form.

Recently Smaers et al. [31] presented an analysis that shows multiple periods of changes in brain and body allometry across mammalian evolution, and McCurry et al. [32] added data on mysticetes to study brain scaling evolution in cetaceans (especially during the Eocene and Oligocene), would likely be refined with our improved dataset.

It is beyond the scope of this paper to investigate how the pattern of brain size evolution in cetaceans correlates with predictions of the causal hypotheses listed in the introduction. We believe that it is too early to do so. Most authors have based the trajectory of Eocene brain size evolution on five to six specimens most of which are basilosaurids. Of the four families lower on the phylogenetic tree than basilosaurids, only two are represented. Gingerich [8] noted EQ increases in the cetaceans during the Eocene, and we concur. We here add a second remingtonocetid to the sample, but there are still no data for pakicetids and ambulocetids. CT-scanning of additional specimens will continue to refine the pattern that we documented, and combined with deeper morphological study, should allow the building of a stronger foundation to test the evolutionary hypotheses.

## Supporting information

S1 Text Detailed discussion of Eocene cetacean brain and body size data and specimens. (DOCX)

S1 File S1, S2 Datas and S1, S2 Figs. S1 Data, Phylogeny used in brain mass and endocranial volume PGLS regression; S2 Data, Phylogeny used in OCW and body mass PGLS regression; S1 Fig, Phylogeny of Upham et al. (2019) used in our brain mass and endocranial volume PGLS regression; S2 Fig, Phylogeny of McGowen et al. (2020) used in OCW and body mass PGLS regression.
(DOCX)

S2 File S1 Table, extant brain mass and endocranial volumes used in analysis; S2 Table, extant cetacean Occipital Condyle Width (OCW) and body masses used in analysis; S3 Table, fossil cetacean brain and body mass and EQ estimates; S4 Table, results of ANOVA and HSD. (DOCX)
