# Supplementary material for: The pattern of brain-size change in the early evolution of cetaceans
Source: PLoS One. 2021 Sep 28;16(9):e0257803. doi: 10.1371/journal.pone.0257803 (PMC8478358; doi:10.1371/journal.pone.0257803)
Supplement: S1 Text — (DOCX) [file pone.0257803.s001.docx]

**S1 Text. Detailed discussion of Eocene cetacean brain and body mass data and specimens.**

We compiled data for archaeocetes from the primary literature to ensure accuracy, verify the context, evaluate assumptions made in the original brain and body mass estimates, and to track the specific specimen, or specimens examined. Table 2 in the main text summarizes the data we include in this study, and we justify these choices here. Gingerich (2016) provides a comprehensive examination of body and brain mass estimations for most Eocene whales and tracks the appearance of the data through the literature. Therefor we do not discuss every occurrence of the specimens through the literature and focus on aspects of the data relevant to our study.

**Basilosauridae**

***Dorudon atrox***

Uhen (1996p.574) reported an OCW of 126 mm for an adult *Dorudon atrox* specimen (UM 101222) and from this measurement we estimate a body mass of 1023 kg using our regression based equation of OCW and body mass for extant cetaceans. Uhen (1996) p.359 first estimated the body mass at 2700 kg based on a reconstructed body length of 485 cm, based on UM 101222 and another adult specimen UM 101215, using a body length-to-mass equation derived from extant cetaceans (Uhen, 1996). Uhen (2004) p.124 revised this estimate to 2240 kg using a new length-to-body mass equation; this estimate was followed by Marino et al. (2004) and Boessenecker et al. (2017).

Gingerich (1998) p.436, using a regression on vertebral elements from UM 101222, and two additional specimens (UM 97506 and UM 101215), estimated a body mass of 1140 kg. Gingerich (2016)p.27 subsequently revised it to 1126 kg. In addition, Gingerich (2016) estimated a body mass of 1118 kg using Uhen’s (1996) reported body length of 485 cm, and employing a new length-to-body mass equation based on a new dataset of extant cetaceans. Our body mass estimate of 1023 kg is similar to those Gingerich (1998, 2016), but is approximately half of the Uhen (2004) estimate used by Marino et al. (2004) and Boessenecker et al. (2017).

Based on CT scans of reported in the literature (Uhen, 1996, 2004; Marino et al., 2000), the endocranial volume of *D. atrox* (UM 101222) is 1173 cm^3^. Marino et al. (2004) p.1249-1250 reports the endocranial volume, which they equate with brain mass, of this specimen (UM 101222) as 1185.4 cm^3^, although Boessenecker et al. (2017) gives, also for the same specimen, an endocranial volume of 1197.4 cm^3^. The basis for the minor variations in these numbers is unclear. Gingerich (1998) p.439 reports that “University of Michigan field parties” collected endocasts with an average volume of 1200 cm^3^ for *D. atrox*, although the specifics of the measurements, or which specimens were utilized is unclear.

Marino et al. (2000) and Uhen (1996, 2004) assume that the endocranial rete occupies 19.5 percent of the endocranial volume and using that assumption estimate a brain volume of 944.3 cm^3^, which they equate this with a brain mass of 944.3 g. Marino et al. (2004), for the same specimen, and possibly in error, made no correction for rete volume or specific gravity, and estimated the brain mass at 1185.4 g, equating the raw endocranial volume as brain mass. Boessenecker et al. (2017), accounting for a rete and brain density, estimated a brain mass of 931.4 g from an endocranial volume of 1197.4 cm^3^. Using our correction for adnexa/non-neural tissue based on an endocranial volume of 1173 cm^3^ from UM 101222 yields a brain mass of 883 g.

***Saghacetus osiris***

Jerison (1978) using body length, and Gingerich (1998) using vertebral sizes, independently estimated the body mass of *S. osiris* to be 350 kg (Jerison (1978) examined BMNH 10228 and Gingerich (1998) examined BMNH 10228 and UM97550), a figure used by subsequent authors (Marino et al., 2004; Boessenecker et al., 2017). Gingerich (2016) (his Table 1) lists more than one *S. osiris* specimen (UM 97550), presumably he used this specimen to get body mass (based on Gingerich (2007) fig. 4 p.368, indicating the specimen included vertebrae. Kellogg (1936) p.246 published an OCW of 91.8 mm for BMNH 10228 and we estimate a body mass of 379 kg using our OCW to body mass regression equation.

Dart (1923) p.634 gives an endocranial volume for *S. osiris* of 480 cm^3^ based on an artificial endocranial cast of BMNH 10228, it should be noted that Kellogg (1936) (p. 198) indicated the “anterior portions of the olfactory peduncles” and “anterior portions of the trigeminal apparatus” are missing from the specimen. Gingerich (1998) p.439 gives an average endocranial volume of 485 cm^3^ for unspecified University of Michigan *S. osiris* specimens, and after applying the 20 percent rete volume correction of Uhen (1996) and Marino et al. (2000), estimates a brain volume of 485 cm^3^ which he equates with a mass of 388 g, a figure which Marino et al. (2004) and others follow. Boessenecker et al. (2017) uses the average endocranial volume (485 cm^3^) given by Gingerich (1998) p.439, applies a 20% rete volume reduction in conjunction with a brain tissue density correction and estimates brain mass at 373 g. Using our correction, we estimate a brain mass of 383 g, based on a raw endocranial volume of 480 cm^3^ given by Dart (1923).

***Zygorhiza kochii***

We include two specimens of *Zygorhiza kochii* in our study. The first specimen, FMNH PM-459, has an OCW of 120 mm (Gingerich, 2015) p.168 and from this we estimate a body mass of 877 kg. For the same specimen, Gingerich (2015) p.185 estimated body mass of 932 kg (based on vertebral measurements). Gingerich (2015) made a second estimate based on vertebral measurements of this species reported in Kellogg (1936); these measurements were acquired from a more complete specimen (presumably USNM 4679 and/or USNM 11962), and from these specimens he estimates a body mass of 998 kg, a figure adopted by Boessenecker et al. (2017) and used in Gingerich (2016). This estimate is in general agreement with our body estimate of 877 kg.

Gingerich (2015) p. 179 reports an endocranial volume of 1189 cm^3^ for FMNH PM-459 obtained from a laser scan of an artificial endocranial cast (UM 116960 which is the specimen number for one of the artificial endocasts of FMNH PM-459). Following Marino et al. (2000), Gingerich (2015) assumes a rete volume of 19.5% and estimates a brain volume of 957 cm^3^ with a corresponding mass of 957 g which he rounds to 960 g. From the raw endocranial volume given by Gingerich (2015) and applying our correction for the presence of non-neural material, we estimate a brain mass of 894 g.

The second specimen of *Zygorhiza kochii* (USNM 16639) we include*,* has likely been confused with another specimen of the same species, USNM 16638. This uncertainty may stem from Kellogg (1936) in which these two specimens were examined, but had not yet been assigned museum numbers which postdate the work of Kellogg. In the text, Kellogg (1936), simply refers to them collectively as the “Millsaps College Museum specimens”, sometimes distinguishing between the “juvenile” and “adult specimen”. Kellogg (1936) initially describes all the examined material in a sequence of numbered paragraphs (Kellogg, (1936): p.105-106), in which the “Millsaps College Museum specimens” appear as: specimen, *19,* which possessed deciduous dentition, and one as another, *20,* which is listed as an adult. Unfortunately, Kellogg does not use these informal paragraph/specimen numbers again in the text. The specimen figured as USNM 16639 by Marino et al. (2000), figure 2) appears, based on similarity with photographs of the specimen in the USNM online catalog (http://n2t.net/ark:/65665/3b2b951f2-81ad-484f-a429-ff1dccf5206b) to be that of USNM 16638, not 16639. In addition, the specimen number (USNM 16638) is inked on the specimen and is visible in the photographs in the catalog entry.

To estimate body mass, we use the OCW of 112 mm, listed as Millsaps College Museum *adult* by Kellogg (1936), Table 62, p.246-247), which we presume is now the specimen cataloged as USNM 16639. Using this OCW we estimate a body mass of 707 kg. Marino et al. (2000) estimated the body mass of *Zygorhiza kochii* based on a reconstructed body length using the same length-to-body mass equation they used for the *Dorudon atrox* specimens (based on extant cetacean body length to body mass relationship). Their body mass estimate of 3351 kg originates from a body length estimate of 520 cm which was derived from a third *Zygorhiza kochii* specimen (USNM 11962), which Marino et al. (2000) attribute to Kellogg (1936). We did not specifically find that body length in Kellogg, but it could be based on reported vertebral and skull length measurements. Kellogg (1936):p.130) does give a body length estimate of 493 cm for *Zygorhiza kochii,* based on a composite reconstruction from multiple specimens which included the cranium of USNM 11962. Marino et al. (2004) give a body mass of 2040 kg for USNM 16638, although the origins of the second estimate are unclear. Using the new length-to mass-equation given by Uhen (2004) would give a mass of 2752 kg, not 2040 kg. Using the body length of 493 cm given by Kellogg and the equation of Uhen would give a mass of 2349 kg. Boessenecker et al. (2017) used the 998 kg estimate of Gingerich (2015) for both *Z. kochii* specimens he included (USNM 16638 and FMNH PM-459).

Marino et al. (2000) report endocranial volumes for both USNM 16638 and 16639 based on CT scans of the specimens. We used their endocranial volume for the specimen with the larger cranial volume (917 vs. 751 cm^3^) which Marino at al. (2000) listed as USNM 16638, although we presume the measurement is actually from USNM 16639, and from this we estimate a brain mass of 701 g. Using the larger of the two volumes, we can assume the number came from the adult Millsaps College Museum specimen. Marino et al. (2000) gives the endocranial volumes 917 and 751 cm^3^ for USNM 16638 and 16639 and after application of the 19% rete volume correction, give corresponding brain mass estimates of 732.8 and 604.6 kg. Marino et al. (2004) reports the brain volume as 800.8 cm^3^ for USNM 16638, although it is unclear how or where that number originated. Boessenecker et al. (2017) lists the endocranial volume of USNM 16638 as 876.8 cm^3^ (although the origin of the raw endocranial volume is unclear), and after correcting for rete and brain tissue density, estimates a brain mass of 702.3 g.

***Basilosaurus cetoides***

Although we do not include *Basilosaurus cetoides* in our dataset for reasons discussed below, data for these specimens has entered the literature and should be examined. Marino et al. (2004) (table 1 p.1249-1250) lists data for two *B. cetoides* specimens, the first of which is attributed to Gingerich (1998) and the second to USNM 11121. The first listing for *B. cetoides*, attributed to Gingerich (1998), is likely a labeling error, and the intent was for this line in the table to represent the *B. isis* reported by Gingerich (1998) rather than *B. cetoides*. This first entry labeled for *B. cetoides* in Marino et al. (2004) lists a body mass of 6480 kg which is the mass given for *Basilosaurus isis* in Gingerich (1998) p.440, the brain mass for this specimen is listed as 2240 g, which is exactly 80% of the 2800 cm^3^ endocranial volume that Gingerich (1998) p.439, gives for *B. isis*, Gingerich (1998, 2016) never published an endocranial volume for *B. cetoides* that we are aware of. Unfortunately, this error has propagated in the literature undetected and the data was incorporated by Manger (2006) and Montgomery et al. (2013).

Marino et al. (2004) (table 1, p.1249-1250) includes a second entry for *B. cetoides* based on USNM 11121 with an estimated body mass of 730.8 kg. For Eocene fossil whales, Marino et al. (2004) generally followed the body mass estimates of Gingerich (1998) or Uhen (2004). Gingerich, to the best of our knowledge, has not published a body mass estimate for this particular specimen (USNM 11121) and Marino et al. (2004) does not use the body mass estimate of 5840 kg that Gingerich (1998) p.436 gives for *B. cetoides* (based on USNM 4674 and 4675). Thus, the origin of the body size estimate of Marino et al. (2004) is not immediately clear. It seems probable that the Mario et al. (2004) body mass estimate of 730.8 kg could be based on the OCW to body mass equation (given by Boessenecker et al. (2017)) to estimate body mass in post-Eocene whales. As we have done for the post-Eocene whales, we reworked the equation of Boessenecker et al. (2017) to calculate the OCW from the body mass estimate. Using that approach, we get a calculated OCW of 141.9 mm which is remarkably close the OCW value that we measured from published images of USNM 11121 (Marino et al., 2003). Using the back calculated OCW from the Marino et al. (2004) body mass estimate, we would estimate (using our equation) a body mass of 1484 kg, which is more than double the estimate of Marino et al. (2004). Gingerich (1998) p.436 estimated the body mass of *B. cetoides* using data from two specimens (USNM 4674 and 4675) at 5840 kg, although Gingerich (2016) concluded that his original estimates were questionable given the unique vertebral morphology of the genus *Basilosaurus*. Kellogg (1936) p.246-247 gives an OCW of 144.8 for USNM 4674 (one of the *B. cetoides* specimens used by Gingerich to estimate body mass) and using that number we would estimate a body mass of 1581 kg, less than 50% of Gingerich’s estimate.

Marino et al. (2004) estimates the brain mass of USNM 11121 as 302.8 g. If we assume Marino et al. (2004) accounted for a rete as they did for most of the basilosaurids, the raw endocranial volume would have been 378.8 cm^3^, and from that we would estimate the brain mass at 309 g, only marginally larger than Marino’s estimate. Although USNM 11121 is a fragmentary specimen and is missing one occipital condyle, our estimate of its OCW are roughly similar USNM 4674 (141.9 mm and 144.8 mm respectively). Given the skull of USNM 4674 is over a meter in length and half a meter wide, an endocranial volume of 378.8 cm^3^ seems implausibly small. Given this apparent anomalously small endocranial volume, and uncertainties in the body mass estimate for *Basilosaurus* as discussed by Gingerich (2016), we currently exclude *B. cetoides* from our dataset.

***Basilosaurus isis***

Gingerich (1998) estimates the body mass of *Basilosaurus isis* as 6480 kg using vertebral elements (three specimens are listed, UM 94800, UM 94801, and UM 97503). Gingerich (2016) notes that both *B. isis* and *B. cetoides* have altered vertebral geometry, and suggests body mass estimates based on vertebral regressions are likely to underestimate body mass, while estimates based on body length are likely overestimates and thus concluding that the current *Basilosaurus* body mass estimates are unreliable. Without published OCW values we cannot make our own body mass estimates for UM 94800, 94801, and 97503, although it is also unclear if some these specimens were used by Gingerich (1998) to ascertain endocranial volume. Kellogg (1936) gives OCW values for two specimens of *B. isis*, Stuttgart 11787 and AMNH 14381, with OCW of 138.5 mm and 124.2 mm respectively, these would correspond to body masses of 1375 kg and 977 kg using our equation, both of which are significantly lower than the Gingerich (1998) estimate of 6480 kg.

Gingerich (1998) p.439 reports an average endocranial volume for *B. isis* as 2800 cm^3^, based on specimens found by “University of Michigan filed parties working in Egypt,” although specimen numbers are not reported. Gingerich makes a 20% volume correction for the rete, and estimates brain mass as 2520 g. Although 2520 is a 10% reduction, a 20% rete correction would result in a brain volume of 2240 cm^3^ , which was likely the original intent of Gingerich (1998). For reasons similar to those discussed for *B. cetoides,* we currently exclude *B. isis* from our dataset.

**Protocetidae**

***Rodhocetus kasranii***

The only protocetid whale for which we have data is *Rodhocetus kasranii*, based on an OCW measurement (88.7 mm) of GSP-UM 3012 (Uhen personal communication, 2020) we estimate a body mass of 340 kg. Gingerich (1998) p.434 examined two specimens of *Rodhocetus kasranii* (GSP-UM 1853 and 3012) and estimated a body mass of 590 kg using vertebral measurements, presumably from the holotype (GSP-UM 3012) which included associated postcranial material. It unclear which of the two specimens, or both, was measured to get the reported endocranial volume given by Gingerich (1998). Given the assumption that GSP-UM 3012 provided the postcranial material used by Gingerich to estimate body mass, it is reasonable to assume the brain mass estimate of Gingerich is taken from GSP-UM 1853 which appears to consist of only of a cranium (Gingerich et al., 1993). Cranial measurement given by Hulbert et al. (1998) of GSP-UM 3012 and GSP-UM 1853 indicate the specimens are of similar size, and thus our body size estimate based on GSP-UM 3012 should be a reasonable proxy for body size even if the endocranial volume reported by Gingerich is from GSP-UM 1853. Marino et al. (2004) p.1249-1250 report a body mass of 290 kg for GSP-UM 3012, although the origin of this number is unclear. Marino et al. (2004) generally used the body mass estimates of Gingerich (1998) for the Eocene whales (which Gingerich (1998) reported as 590 kg), as 290 g was the endocranial volume reported by Gingerich (1998), a simple transcription error between the brain and body masses may have occurred.

Gingerich (1998) p.434 reports an endocranial volume of 290 cm^3^ for *R. kasranii,* although both GSP-UM 1853 and 3012 are listed in the figure, it is unclear which of those specimens, or a combination, was the basis for the endocranial volume. It is also unclear how exactly the endocranial volume was measured. The figure caption states that “brain masses are estimated from endocranial casts associated with skulls” (Gingerich, 1998) p434-435,fig 4 caption. Bajpai et al. (1996) p.582 report an endocranial volume 290 cm^3^ for an isolated endocranial cast of *Indocetus* (VPL 1018, *Indocetus* sp., cf. *I. ramani*. *Rodhocetus* and *Indocetus* are very similar, the type description of *Rodhocetus* (Gingerich et al., 1994) lists four diagnostic differences with *Indocetus*, but three of these were based on specimens misattributed to *Indocetus* (Gingerich et al., 1995). The one remaining difference (convex posterior surface of the exoccipital), is not listed as diagnostic by Gingerich (1995), and that paper does not list any differences between these genera in the diagnosis, but states (p. 300): ‘*Rodhocetus* and *Protocetus* differ in the degree of caudalization of the sacrum, and it is reasonable to expect that later *Indocetus* probably differed even more. However, this remains to be substantiated by discovery of the sacrum and pelvis of *Indocetus*’. Moran et al. (2015) described that fusion of vertebrae in cetaceans happens as part of aging, late in life. GSP-UM 1853 (one of the specimens Gingerich used for *Rodhocetus* and the specimen from which we estimate body mass) had been preciously assigned to *Indocetus ramani* and was subsequently referred to *Rodhocetus kasranii* when Gingerich erected the genus and species (Gingerich et al., 1995). *Rodhocetus kasranii* may very well be a junior synonym of *Indocetus ramani* Gingerich does not make a correction for a rete, citing Bajpai et al. (1996) who note that the two endocasts they examined, and referred to *Indocetus*, did not possess the large “endocranial vasculature” of the later Eocene cetaceans from Egypt. Marino et al. (2004) p.1249-1250, citing Gingerich (1998) (for GSP-UM 3012), report a brain mass of 291 g, one gram more that Gingerich (1998) reported. Boessenecker et al. (2017) used an endocranial volume of 291 cm^3^, and correcting for brain density tissue density and applying their non-rete specific correction for adnexa based on extant cetaceans, estimate a brain mass of 272.7 g. Using the endocranial volume reported by Gingerich (1998) of 290 cm^3^_,_ we estimate a brain mass of 240 g.

**Remingtonocetidae**

***Dalanistes ahmedi***

From the width of the occipital condyles (104.8 mm) of the type specimen of *Dalanistes ahmedi* GSP-UM 3106 (Uhen, personal communication, 2020), we estimate a body mass of 574 kg. Gingerich (1998) p.434 examined four specimens of *D. ahmedi* (GSP-UM 3099, 3106, 3165, and NHML 50719) and using measurements of vertebral elements estimated a body mass of 750 kg, and Marino et al. (2004) and Boessenecker et al. (2017) have followed the original 750 kg estimate of Gingerich (1998).

Gingerich (1998) p.434 gives an endocranial volume of 400 cm^3^ for *Dalanistes ahmedi*, although it is unclear from which of the four specimens listed (GSP-UM 3099, 3106, 3165, and NHML 50719) the value is derived. Gingerich et al. (1995) p.325 observes that *D. ahmedi* lacks the large rete present in the basilosaurids and therefore does not make a correction for rete volume. Gingerich (1998) estimated brain mass as 400 g, which was followed by Marino et al. (2004), while Boessenecker et al. (2017) correct for adnexa and brain tissue density and estimated a mass of 372.2 g. Using the endocranial volume of 400 cm^3^ we estimate a brain mass of 323 g.

***Remingtonocetus harudiensis***

Bajpai et al. (2011) p.716 give an endocranial volume of 253 cm^3^ for *R. harudiensis* (IITR-SB 2770). This specimen has not always been included in compiled fossil brain datasets, likely because Bajpai et al. (2011) gave a wide range for the body mass estimate. The OCW for *R. harudiensis* (IITR-SB 2770) of 87 mm is reported in Bajpai et al., (2011) and from this we estimate a body mass of 320 kg.

The endocranial volume of 253 cm^3^ for *Remingtonocetus harudiensis* comes from a virtual endocast bases on CT scan of IITR-SB 2770, published in Bajpai et al., (2011). From this endocranial volume we estimate a brain mass of 211 g.

**S1 Specimens References**

Bajpai, S., Thewissen, J.G.M., and Sahni, A., 1996, *Indocetus* (Cetacea, Mammalia) endocasts from Kachchh (India): Journal of Vertebrate Paleontology, v. 16, p. 582–584.

Bajpai, S., Thewissen, J.G.M., and Conley, R.W., 2011, Cranial anatomy of middle Eocene *Remingtonocetus* (Cetacea, Mammalia) from Kutch, India: Journal of Paleontology, v. 85, p. 703–718.

Boessenecker, R.W., Ahmed, E., and Geisler, J.H., 2017, New records of the dolphin *Albertocetus meffordorum* (Odontoceti: Xenorophidae) from the lower Oligocene of South Carolina: Encephalization, sensory anatomy, postcranial morphology, and ontogeny of early odontocetes: PLoS ONE, v. 12, p. e0186476.

Dart, R.A., and Andrews, C.W., 1923, The brain of the Zeuglodonlidæ (Cetacea): Proceedings of the Zoological Society of London, v. 93, p. 615–654.

Gingerich, P.D., 1998, Paleobiological perspectives on Mesonychia, Archaeoceti, and the origin of whales; p. 423–449. *In* J.G.M. Thewissen (ed.), The emergence of whales. Springer US, Boston, MA.

Gingerich, P.D., 2007, *Stromerius nidensis*, new archaeocete (Mammalia, Cetacea) from the upper Eocene Qasr el-Sagha Formation, Fayum, Egypt: .

Gingerich, P.D., 2015, New partial skeleton and relative brain size in the late Eocene Archaeocete Zygorhiza kochii (Mammalia, Cetacea) from the Pachuta Marl of Alabama, with a note on contemporaneous Pontogeneus brachyspondylus: Contributions from the Museum of Paleontology, University of Michigan, v. 32, p. 161–188.

Gingerich, P.D., 2016, Body weight and relative brain size (encephalization) in Eocene Archaeoceti (Cetacea): Journal of Mammalian Evolution, v. 23, p. 17–31.

Gingerich, P.D., Arif, M., and Clyde, W.C., 1995, New Archaeocetes (Mammalia, Cetacea) from the Middle Eocene Domanda Formation of Sulaiman Range, Punjab (Pakistan): Contributions from the Museum of Paleontology, University of Michigan, v. 29, p. 291–330.

Gingerich, P.D., Raza, S.M., Arif, M., Anwar, M., and Zhou, X., 1993, Partial skeletons of *Indocetus ramani* (Mammalia, Cetacea) from the lower middle Eocene Domanda Shale in the Sulaiman range of Punjab (Pakistan): .

Hulbert, R.C., Petkewich, R.M., Bishop, G.A., Bukry, D., and Aleshire, D.P., 1998, A New Middle Eocene Protocetid Whale (Mammalia: Cetacea: Archaeoceti) and Associated Biota From Georgia: 907–927 p.

Jerison, H.J., 1978, Brain and intelligence in whales: Whales and Whaling, v. 2, p. 159–197.

Kellogg, R., 1936, A review of the Archaeoceti: Carnegie Institution of Washington Publication, v. 482, p. 1–366.

Manger, P.R., 2006, An examination of cetacean brain structure with a novel hypothesis correlating thermogenesis to the evolution of a big brain: Biological Reviews of the Cambridge Philosophical Society, v. 81, p. 293–338.

Marino, L., Mcshea, D.W., and Uhen, M.D., 2004, Origin and evolution of large brains in toothed whales: Anatomical Record - Part A Discoveries in Molecular, Cellular, and Evolutionary Biology, v. 281, p. 1247–1255.

Marino, L., Uhen, M.D., Pyenson, N.D., and Frohlich, B., 2003, Reconstructing cetacean brain evolution using computed tomography: Anatomical Record - Part B New Anatomist, v. 272, p. 107–117.

Marino, L., Uhen, M.D., Frohlich, B., Aldag, J.M., Blane, C., Bohaska, D., and Whitmore, F.C., 2000, Endocranial volume of mid-late Eocene archaeocetes (order: Cetacea) revealed by computed tomography: Implications for cetacean brain evolution: Journal of Mammalian Evolution, v. 7, p. 81–94.

Montgomery, S.H., Geisler, J.H., McGowen, M.R., Fox, C., Marino, L., and Gatesy, J., 2013, The evolutionary history of cetacean brain and body size: Evolution, v. 67, p. 3339–3353.

Moran, M.M., Bajpai, S., George, J.C., Suydam, R.S., Usip, S., and Thewissen, J.G.M., 2015, Intervertebral and Epiphyseal Fusion in the Postnatal Ontogeny of Cetaceans and Terrestrial Mammals: Journal of Mammalian Evolution, v. 22, p. 93–109.

Uhen, M.D., 1996, Dorudon atrox (Mammalia, Cetacea): form, function, and phylogenetic relationships of an Archaeocete from the late middle Eocene of Egypt: University of Michigan, Ann Arbor, 608 p.

Uhen, M.D., 2004, Form, function, and anatomy of Dorudon atrox (Mammalia, Cetacea): an Archaeocete from the middle to late Eocene of Egypt; p. 222. *In* The University of Michigan Museum of Paleontology Papers on Paleontology. v. 34. Museum of Paleontology, The University of Michigan.
