# Supplementary material for: The pattern of brain-size change in the early evolution of cetaceans
Source: PLoS One. 2021 Sep 28;16(9):e0257803. doi: 10.1371/journal.pone.0257803 (PMC8478358; doi:10.1371/journal.pone.0257803)
Supplement: S1 File — S1 Data, Phylogeny used in brain mass and endocranial volume PGLS regression; S2 Data, Phylogeny used in OCW and body mass PGLS regression; S1 Fig, Phylogeny of Upham et al. (2019) used in our brain mass and endocranial volume PGLS regression; S2 Fig, Phylogeny of McGowen et al. (2020) used in OCW and body mass PGLS regression. (DOCX) [file pone.0257803.s002.docx]

**S1- S2 Data and S1-S2 Figures**

**S1 Data, Phylogeny used in brain mass and endocranial volume PGLS regression.**

For the brain mass and endocranial volume PGLS regression we use the node dated phylogeny of Upham et al. (2019) found in their supplementary data (Upham et al. (2019):S3 Data deposited in DRYAD [doi.org/10.5061/dryad.tb03d03](https://doi.org/10.5061/dryad.tb03d03) file: “MamPhy_fullPosterior_BDvr_Completed_5911sp_topoCons_FBDasZhouEtAl_MCC_v2_target.tre”

Tree file trimmed for taxa included in our study:

((Tamandua_tetradactyla_MYRMECOPHAGIDAE_PILOSA:13.9121874,Myrmecophaga_tridactyla_MYRMECOPHAGIDAE_PILOSA:13.9121874):79.95950797,((((((Cerdocyon_thous_CANIDAE_CARNIVORA:2.575726911,(Pseudalopex_culpaeus_CANIDAE_CARNIVORA:0.5193918774,Pseudalopex_gymnocercus_CANIDAE_CARNIVORA:0.5193918774):2.056335033):0.3661537449,Pseudalopex_sechurae_CANIDAE_CARNIVORA:2.941880656):2.312657237,(Canis_latrans_CANIDAE_CARNIVORA:1.749938172,(Canis_aureus_CANIDAE_CARNIVORA:1.422163804,Canis_lupus_CANIDAE_CARNIVORA:1.422163804):0.3277743681):3.50459972):33.4659687,((Mustela_nivalis_MUSTELIDAE_CARNIVORA:3.91938026,Mustela_erminea_MUSTELIDAE_CARNIVORA:3.91938026):6.802923195,(Martes_foina_MUSTELIDAE_CARNIVORA:7.123500753,Eira_barbara_MUSTELIDAE_CARNIVORA:7.123500753):3.598802702):27.99820314):28.24188274,((Equus_africanus_EQUIDAE_PERISSODACTYLA:5.599832557,(Equus_ferus_EQUIDAE_PERISSODACTYLA:0.8567015576,Equus_caballus_EQUIDAE_PERISSODACTYLA:0.8567015576):4.743130999):44.29023015,(Lama_guanicoe_CAMELIDAE_CETARTIODACTYLA:49.89001453,(Sus_scrofa_SUIDAE_CETARTIODACTYLA:45.40303369,((Balaena_mysticetus_BALAENIDAE_CETARTIODACTYLA:15.05180427,(Delphinapterus_leucas_MONODONTIDAE_CETARTIODACTYLA:7.559012865,Tursiops_truncatus_DELPHINIDAE_CETARTIODACTYLA:7.559012865):7.4927914):25.25741611,(Bos_taurus_BOVIDAE_CETARTIODACTYLA:11.31913453,(Capra_hircus_BOVIDAE_CETARTIODACTYLA:0.5283643083,Capra_aegagrus_BOVIDAE_CETARTIODACTYLA:0.5283643083):10.79077022):28.99008585):5.093813312):4.486980839):4.817947757e-05):17.07232663):14.23571837,(Homo_sapiens_HOMINIDAE_PRIMATES:75.29769309,Oryctolagus_cuniculus_LEPORIDAE_LAGOMORPHA:75.29769309):5.900414625):12.67358765);

**S2 Data, Phylogeny used in OCW and body mass PGLS regression.**

For the OCW and body mass PGLS regression we use the node dated phylogeny of McGowen et al. (2020) found in their supplementary data deposited in DRYAD (McGowen et al. (2019) <https://doi.org/10.5061/dryad.jq40b0f>

“FigTree_parts_6_mcmctree_AR.tre ”.

Abbreviations used in their tree tips were matched the full species names which were found in their ASTRAL tree which were in turn matched with our data as follow: In their tree *Kogia brevirostris* is matched with *Kogia breviceps* in our data as the names are synonymous. Their *Mesoplodon grayii* is matched with our *Mesoplodon grayi,* *Mesoplodon layardi* with our *Mesoplodon layardii*, their *Mesoplodon gingkodens* with our *Mesoplodon ginkgodens*; and our *Indopacetus pacificus* was matched with their *Mesoplodon bowdoini* based on the phylogeny of Dalebout et al. (2002).

Tree file trimmed for taxa included in our study:

((((((Tursiops_truncatus:0.0812955,(Peponocephala_electra:0.02258,Feresa_attenuata:0.02258):0.0587155):0.01113233333,Lagenorhynchus_obliquidens:0.09242783333):0.1054180417,(Phocoena_dioptrica:0.042876,Phocoena_phocoena:0.042876):0.154969875):0.1148190801,((((((((Mesoplodon_perrini:0.030346,Mesoplodon_peruvianus:0.030346):0.008033,Mesoplodon_densirostris:0.038379):0.0087855,Mesoplodon_grayi:0.0471645):0.005086875,Mesoplodon_hectori:0.052251375):0.01211759167,((Mesoplodon_carlhubbsi:0.046408,Indopacetus_pacificus:0.046408):0.0082795,Mesoplodon_layardii:0.0546875):0.009681466667):0.007405095833,((Mesoplodon_mirus:0.048993,Mesoplodon_ginkgodens:0.048993):0.017569,Mesoplodon_bidens:0.066562):0.0052120625):0.04023998295,Ziphius_cavirostris:0.1120140455):0.04408699621,Berardius_bairdii:0.1561010417):0.1565639135):0.02867304487,(Kogia_sima:0.077469,Kogia_breviceps:0.077469):0.263869):0.02590558333,((((Balaenoptera_edeni:0.044996,Balaenoptera_borealis:0.044996):0.067105,Balaenoptera_musculus:0.112101):0.045309,(Balaenoptera_bonaerensis:0.075959,Balaenoptera_acutorostrata:0.075959):0.081451):0.0998615,Eubalaena_glacialis:0.2572715):0.1099720833);

**S1 Figure**

**S1 Figure**, Phylogeny of Upham et al. (2019) used in our brain mass and endocranial volume PGLS regression.

**S2 Figure**

**S2 Figure**, Phylogeny of McGowen et al. (2020) used in OCW and body mass PGLS regression.

**References S1- S2 Data and S1-S2 Figures**

Dalebout, M.L., Mead, J.G., Baker, C.S., Baker, A.N., and Van Helden, A.L., 2002, A new species of beaked whale *Mesoplodon perrini* sp. n. (Cetacea: Ziphiidae) discovered through phylogenetic analyses of mitochondrial DNA sequences: Marine Mammal Science, v. 18, p. 577–608.

McGowen, M.R., Tsagkogeorga, G., Álvarez-Carretero, S., Dos Reis, M., Struebig, M., Deaville, R., Jepson, P.D., Jarman, S., Polanowski, A., Morin, P.A., and Rossiter, S.J., 2020, Phylogenomic Resolution of the Cetacean Tree of Life Using Target Sequence Capture: Systematic Biology, v. 69, p. 479–501.

McGowen, Michael et al. (2019), Data from: Phylogenomic resolution of the cetacean tree of life using target sequence capture, Dryad, Dataset, <https://doi.org/10.5061/dryad.jq40b0f>

Upham, N.S., Esselstyn, J.A., and Jetz, W., 2019, Inferring the Mammal Tree: Species-Level Sets of Phylogenies for Questions in Ecology, Evolution, and Conservation: 1–44 p.

Upham, Nathan S.; Esselstyn, Jacob A.; Jetz, Walter (2019), Inferring the mammal tree: Species-level sets of phylogenies for questions in ecology, evolution, and conservation, Dryad, Dataset, <https://doi.org/10.5061/dryad.tb03d03>
