# Supplementary material for: The pattern of brain-size change in the early evolution of cetaceans
Source: PLoS One. 2021 Sep 28;16(9):e0257803. doi: 10.1371/journal.pone.0257803 (PMC8478358; doi:10.1371/journal.pone.0257803)
Supplement: S2 File — (DOCX) [file pone.0257803.s003.docx]

**S1, S2, S3, and S4 Tables**

**S1 Table. Extant brain mass and endocranial volumes used in analysis.**

| **Species** | **Number of specimens** | **Reference** | **Measurement type** | **Notes** | **Brain mass (g)** | **Endocranial volume (cm^3^)** |
| --- | --- | --- | --- | --- | --- | --- |
| *Bos taurus* | 1 | (Köppel, 1898) | mass/vol. |  | 446.00 | 610.00 |
| *Equus asinus* | 23 | (Mobilio, 1915) | mass/vol. |  | 391.89 | 450.00 |
| *Equus caballus* | 39 | (Mobilio, 1915) | mass/vol. |  | 624.47 | 715.57 |
| *Homo sapiens* | 89 | (Reite et al., 2010) | vol./vol. | 3 | 1,327.22 | 1,413.37 |
| *Tursiops truncatus* | 1 | (Ridgway et al., 2016) | vol./vol. | 2 | 1,510.00 | 1,576.92 |
| *Canis aureus* | 32 | (Röhrs and Ebinger, 2001) | mass/vol. | 1 | 65.09 | 69.55 |
| *Canis latrans* | 32 | (Röhrs and Ebinger, 2001) | mass/vol. | 1 | 89.51 | 93.52 |
| *Canis lupus* | 65 | (Röhrs and Ebinger, 2001) | mass/vol. | 1 | 137.39 | 148.94 |
| *Capra hircus* | 36 | (Röhrs and Ebinger, 2001) | mass/vol. | 1 | 120.32 | 134.83 |
| *Cerdocyon thous* | 7 | (Röhrs and Ebinger, 2001) | mass/vol. | 1 | 48.25 | 50.65 |
| *Eira barbara* | 3 | (Röhrs and Ebinger, 2001) | mass/vol. | 1 | 48.06 | 50.34 |
| *Equus przewalskii* | 14 | (Röhrs and Ebinger, 2001) | mass/vol. | 1 | 578.49 | 649.86 |
| *Lama guanacoe* | 20 | (Röhrs and Ebinger, 2001) | mass/vol. | 1 | 265.13 | 277.14 |
| *Lycalopex culpaeus* | 15 | (Röhrs and Ebinger, 2001) | mass/vol. | 1 | 56.63 | 57.29 |
| *Lycalopex gymnocercus* | 56 | (Röhrs and Ebinger, 2001) | mass/vol. | 1 | 41.68 | 41.97 |
| *Lycalopex sechurae* | 4 | (Röhrs and Ebinger, 2001) | mass/vol. | 1 | 33.80 | 33.46 |
| *Martes foina* | 19 | (Röhrs and Ebinger, 2001) | mass/vol. | 1 | 21.41 | 22.45 |
| *Mustela erminea* | 29 | (Röhrs and Ebinger, 2001) | mass/vol. | 1 | 5.41 | 5.40 |
| *Mustela nivalis* | 78 | (Röhrs and Ebinger, 2001) | mass/vol. | 1 | 2.28 | 2.31 |
| *Myrmecophaga tridactyla* | 3 | (Röhrs and Ebinger, 2001) | mass/vol. | 1 | 85.50 | 96.58 |
| *Oryctolagus cuniculus* | 35 | (Röhrs and Ebinger, 2001) | mass/vol. | 1 | 10.57 | 10.45 |
| *Tamandua tetradactyla* | 3 | (Röhrs and Ebinger, 2001) | mass/vol. | 1 | 27.17 | 28.32 |
| *Balaena mysticetus* | 1 | (Thewissen et al., 2011) | mass/vol. | 5 | 2,980 | 8,900 |
| *Delphinapterus leucas* | 1 | (this study) | mass/vol. | 4 | 2,074.00 | 2,528.00 |
| *Capra aegagrus hircus* | 4 | (this study) | mass/vol. | 4 | 109.05 | 139.00 |
| *Sus scrofa* | 2 | (this study) | mass/vol. | 4 | 134.25 | 181.00 |

S1 Table notes:

mass/vol., in the ‘Measurement Type’ column, indicates the brain mass and endocranial volume were recorded directly in mass and volumetric units respectively; vol./vol., indicates that both measurements were volumetric (CT or MRI scan) and the reported brain mass is the specific density of brain tissue multiplied by brain volume. Numbers in the ‘Notes’ column correspond to the following:

1, Röhrs and Ebinger (2001) report brain volume (although they measured mass) which they calculated from mass assuming a brain tissue density of 1.036 g/cm^3^. We multiply their reported brain volumes by 1.036 g/cm^3^ to get brain mass.

2, Ridgway et al. (2016) report brain and adnexa mass, because their reported numbers are derived from a MRI scan, we assume the reported combined mass of adnexa and brain originated as a volume, and therefore we divide the reported mass by 1.04 g/cm^3^ (which is the density of cetacean brains they report) to obtain the endocranial volume.

3, Reite et al. (2010) reported brain and endocranial cranial volumes obtained from MRI scans, we calculate brain mass by multiplying the reported brain volume by 1.036 g/cm^3^.

4, See Table 1 in the main text for specimen specifics.

**S2 Table. Extant cetacean occipital condyle width (OCW) and body masses used in analysis.**

| **Species** | **Reference (age, length, OCW)** | **Body length (cm)** | **OCW (mm)** | **Reported age^1^** | **Assigned age^2^** | **Length at maturity^3^ (cm)** | **reported body mass^4^ (kg)** | **Body mass from body^5^ length (kg)** | **Reference (body mass from length formula)^6^** | **Body mass^7^ (kg)** |
| --- | --- | --- | --- | --- | --- | --- | --- | --- | --- | --- |
| *Balaena mysticetus* | (Nishiwaki and Kasuya, 1970a) | 640 | 275 | J | J | 1158 |  | 4,753.0 | (George, 2009) | 4,753.0 |
| *Balaenoptera acutorostrata* | (Allen, 1916) | 441.96 | 135 | J | J | 670 |  | 923.4 | (Lockyer, 1976) | 923.4 |
| *Balaenoptera acutorostrata* | (Allen, 1916) | 464.82 | 150 | J | J | 670 |  | 1,086.8 | (Lockyer, 1976) | 1,086.8 |
| *Balaenoptera acutorostrata* | (Meirelles et al., 2011) | 700 | 155 | A | A | 670 |  | 4,078.3 | (Lockyer, 1976) | 4,078.3 |
| *Balaenoptera acutorostrata* | (Omura, 1975) | 540 | 154 | J | J | 670 |  | 1,763.8 | (Lockyer, 1976) | 1,763.8 |
| *Balaenoptera acutorostrata* | (Omura, 1975) | 660 | 157 | J | J | 670 |  | 3,372.4 | (Lockyer, 1976) | 3,372.4 |
| *Balaenoptera acutorostrata* | (Secchi et al., 2003) | 343 | 125.7 | J | J | 670 |  | 407.2 | (Lockyer, 1976) | 407.2 |
| *Balaenoptera bonaerensis* | (Arnold et al., 1987) | 710 | 174.9 | A | A |  |  | 4,313.1 | (Ohsumi et al., 1970) | 4,313.1 |
| *Balaenoptera bonaerensis* | (Balensiefer et al., 2014) | 550 | 150 | J | J |  |  | 2,391.2 | (Ohsumi et al., 1970) | 2,391.2 |
| *Balaenoptera bonaerensis* | (Omura, 1975) | 850 | 203 | A | A |  |  | 6,536.5 | (Ohsumi et al., 1970) | 6,536.5 |
| *Balaenoptera bonaerensis* | (Omura, 1975) | 980 | 228 | A | A |  |  | 9,080.7 | (Ohsumi et al., 1970) | 9,080.7 |
| *Balaenoptera bonaerensis* | (Rosel et al., 2016) | 768.1 | 196 | N/A | A |  |  | 5,172.5 | (Ohsumi et al., 1970) | 5,172.5 |
| *Balaenoptera borealis* | (Nishiwaki and Kasuya, 1971) | 1530 | 300 | A | A | 1204 |  | 18,306.9 | (Lockyer, 1976) | 18,306.9 |
| *Balaenoptera borealis* | (Omura, 1959) | 1310.64 | 251 | N/A | A | 1204 |  | 12,569.0 | (Lockyer, 1976) | 12,569.0 |
| *Balaenoptera edeni* | (Omura, 1959) | 1371.6 | 266 | J | J |  |  | 14,037.1 | (Lockyer, 1976) (equation for *Balaenoptera borealis)* | 14,037.1 |
| *Balaenoptera edeni* | (Omura et al., 1981) | 1420 | 285 | A | A |  |  | 15,271.3 | (Lockyer, 1976) (equation for *Balaenoptera borealis)* | 15,271.3 |
| *Balaenoptera edeni* | (Omura et al., 1981) | 1470 | 288 | A | A |  |  | 16,611.0 | (Lockyer, 1976) (equation for *Balaenoptera borealis)* | 16,611.0 |
| *Balaenoptera musculus brevicauda* | (Omura et al., 1970) | 1860 | 365 | A | A |  | 42988 | 43,855.7 | (Lockyer, 1976) | 42,988.0 |
| *Balaenoptera physalus* | (Allen, 1916) | 1463 | 298.4 | N/A | J | 1676 |  | 16,137.5 | (Lockyer, 1976) | 16,137.5 |
| *Berardius arnouxi* | (McCann, 1975) | 271 | 90 | N/A | J | 950 |  | 198.6 | Kasuya et al., 1997 in (Perrin et al., 2005) | 198.6 |
| *Berardius arnouxi* | (McCann, 1975) | 320 | 93 | N/A | J | 1000 |  | 331.4 | Kasuya et al., 1997 in (Perrin et al., 2005) | 331.4 |
| *Berardius arnouxi* | (McCann, 1975) | 870 | 220 | N/A | J | 1000 |  | 7,222.4 | Kasuya et al., 1997 in (Perrin et al., 2005) | 7,222.4 |
| *Berardius bairdii* | (Omura et al., 1955) | 1097 | 238 | A | A | 1000 |  | 14,753.6 | Kasuya et al., 1997 in (Perrin et al., 2005) | 14,753.6 |
| *Berardius bairdii* | (Pike, 1953) | 1013.4 | 246 | A | A | 950 |  | 11,556.6 | Kasuya et al., 1997 in (Perrin et al., 2005) | 11,556.6 |
| *Berardius bairdii* | (Pike, 1953) | 891.5 | 216 | J | J | 1000 |  | 7,786.5 | Kasuya et al., 1997 in (Perrin et al., 2005) | 7,786.5 |
| *Berardius bairdii* | (True, 1910) | 1224.28 | 261 | A | A | 1000 |  | 20,691.0 | Kasuya et al., 1997 in (Perrin et al., 2005) | 20,691.0 |
| *Berardius bairdii* | (True, 1910) | 774.7 | 195 | J | J | 950 |  | 5,051.7 | Kasuya et al., 1997 in (Perrin et al., 2005) | 5,051.7 |
| *Eschrichtius robustus* | (Nishiwaki and Kasuya, 1970b) | 900 | 265 | J | J | 1110 |  | 6,878.4 | (Lockyer, 1976) | 6,878.4 |
| *Eubalaena glacialis* | (Omura et al., 1969) | 1710 | 427 | A | A | 1500 | 67239 | 73,517.3 | (Lockyer, 1976) | 67,239.0 |
| *Eubalaena glacialis* | (Omura et al., 1969) | 1700 | 464 | A | A | 1500 | 65756 | 72,209.7 | (Lockyer, 1976) | 65,756.0 |
| *Eubalaena glacialis* | (Omura et al., 1971) | 1520 | 353 | J | J | 1500 | 48562 | 51,270.0 | (Lockyer, 1976) | 48,562.0 |
| *Eubalaena glacialis* | (Omura et al., 1971) | 1260 | 370 | J | J | 1550 | 28917 | 28,877.2 | (Lockyer, 1976) | 28,917.0 |
| *Eubalaena glacialis* | (Omura, 1958) | 1165 | 399 | J | J | 1550 | 22866 | 22,718.5 | (Lockyer, 1976) | 22,866.0 |
| *Eubalaena glacialis* | (Omura, 1958) | 1240 | 396 | J | J | 1500 | 22247 | 27,497.4 | (Lockyer, 1976) | 22,247.0 |
| *Feresa attenuata* | (Nishiwaki et al., 1965) | 240 | 107 | A | A | 214 | ­– | 181.2 | Ross and Leatherwood (1994) in (Perrin et al., 2005) | 181.2 |
| *Feresa attenuata* | (Nishiwaki et al., 1965) | 217 | 92 | A | A | 214 | – | 135.5 | Ross and Leatherwood (1994) in (Perrin et al., 2005) | 135.5 |
| *Feresa attenuata* | (Nishiwaki et al., 1965) | 215 | 88 | A | A | 214 | – | 131.9 | Ross and Leatherwood (1994) in (Perrin et al., 2005) | 131.9 |
| *Feresa attenuata* | (Nishiwaki et al., 1965) | 229 | 99 | A | A | 214 | – | 158.2 | Ross and Leatherwood (1994) in (Perrin et al., 2005) | 158.2 |
| *Feresa attenuata* | (Nishiwaki et al., 1965) | 223 | 87 | A | A | 214 | – | 146.6 | Ross and Leatherwood (1994) in (Perrin et al., 2005) | 146.6 |
| *Feresa attenuata* | (Nishiwaki et al., 1965) | 214 | 96 | A | A | 214 | – | 130.2 | Ross and Leatherwood (1994) in (Perrin et al., 2005) | 130.2 |
| *Feresa attenuata* | (Nishiwaki et al., 1965) | 225 | 89 | A | A | 214 | – | 150.4 | Ross and Leatherwood (1994) in (Perrin et al., 2005) | 150.4 |
| *Feresa attenuata* | (Nishiwaki et al., 1965) | 225 | 94 | A | A | 214 | – | 150.4 | Ross and Leatherwood (1994) in (Perrin et al., 2005) | 150.4 |
| *Feresa attenuata* | (Nishiwaki et al., 1965) | 221 | 93 | A | A | 214 | – | 142.8 | Ross and Leatherwood (1994) in (Perrin et al., 2005) | 142.8 |
| *Feresa attenuata* | (Nishiwaki et al., 1965) | 227 | 97 | A | A | 214 | 155.8 | 154.3 | Ross and Leatherwood (1994) in (Perrin et al., 2005) | 155.8 |
| *Feresa attenuata* | (Nishiwaki et al., 1965) | 221 | 90 | A | A | 214 | 145.5 | 142.8 | Ross and Leatherwood (1994) in (Perrin et al., 2005) | 145.5 |
| *Feresa attenuata* | (Nishiwaki et al., 1965) | 208 | 93 | A | A | 214 | 110 | 119.9 | Ross and Leatherwood (1994) in (Perrin et al., 2005) | 110.0 |
| *Feresa attenuata* | (Yamada, 1954a) | 235 | 98 | A | A | 214 | – | 170.5 | Ross and Leatherwood (1994) in (Perrin et al., 2005) | 170.5 |
| *Hyperoodon ampullatus* | (Mitchell and Kozicki, 1975) | 615 | 252 | N/A | J | 700 | – | 2,495.6 | (this paper)^8^ | 2,495.6 |
| *Indopacetus pacificus* | (Dalebout et al., 2002) | 596 | 155 | A | A |  | – | 2,272.5 | (this paper)^8^ | 2,272.5 |
| *Indopacetus pacificus* | (Dalebout et al., 2002) | 363 | 126 | J | J |  | – | 517.4 | (this paper)^8^ | 517.4 |
| *Kogia breviceps* | (Kitchener et al., 2012) | 268 | 81.3 | A | A | 266 | – | 297.5 | (this paper)^9^ | 297.5 |
| *Kogia breviceps* | (Kitchener et al., 2012) | 211 | 66.5 | J | J | 270 | – | 149.9 | (this paper)^9^ | 149.9 |
| *Kogia breviceps* | (Layne, 1965) | 201.9 | 72 | J | J | 270 | – | 132.1 | (this paper)^9^ | 132.1 |
| *Kogia breviceps* | (Martins et al., 1985) | 250 | 82 | N/A | J | 266 | – | 243.7 | (this paper)^9^ | 243.7 |
| *Kogia breviceps* | (Omura and Takahashi, 1981) | 248 | 78 | J | J | 270 | – | 238.2 | (this paper)^9^ | 238.2 |
| *Kogia breviceps* | (Omura et al., 1984) | 173.1 | 64 | J | J | 266 | 82.4 | 85.0 | (this paper)^9^ | 82.4 |
| *Kogia breviceps* | (Sanino and Yanez, 1997) | 340 | 100 | N/A | A | 266 | – | 588.5 | (this paper)^9^ | 588.5 |
| *Kogia breviceps* | (Yamada, 1954b) | 222 | 72 | N/A | J | 266 | – | 173.4 | (this paper)^9^ | 173.4 |
| *Kogia simus* | (Chou, 1989) | 256 | 76 | A | A | 210 | – | 260.9 | (this paper)^9^ | 260.9 |
| *Kogia simus* | (Pinedo, 1987) | 249 | 80 | A | A | 210 | – | 240.9 | (this paper)^9^ | 240.9 |
| *Lagenorhynchus obliquidens* | (Sleptsov, 1955) | 221 | 88 | A | A | 173 | – | 143.0 | (Heise, 1997) | 143.0 |
| *Lagenorhynchus obliquidens* | (Sleptsov, 1955) | 204 | 88 | A | A | 173 | – | 114.1 | (Heise, 1997) | 114.1 |
| *Lagenorhynchus obliquidens* | (Sleptsov, 1955) | 200 | 89 | A | A | 176 | – | 107.9 | (Heise, 1997) | 107.9 |
| *Lagenorhynchus obliquidens* | (Sleptsov, 1955) | 220 | 86 | A | A | 173 | – | 141.2 | (Heise, 1997) | 141.2 |
| *Mesoplodon bidens* | (Martín et al., 2011) | 456 | 111 | A | A |  | – | 1,022.0 | (this paper)^8^ | 1,022.0 |
| *Mesoplodon bidens* | (Reiner, 1986) | 483 | 110 | A | A |  | – | 1,213.4 | (this paper)^8^ | 1,213.4 |
| *Mesoplodon bidens* | (Turner, 1882) | 426.72 | 101.6 | A | A |  | – | 838.3 | (this paper)^8^ | 838.3 |
| *Mesoplodon carlhubbsi* | (Moore, 1963) | 518.6 | 130 | A | A |  | – | 1,500.3 | (this paper)^8^ | 1,500.3 |
| *Mesoplodon carlhubbsi* | (Moore, 1963) | 487.6 | 140 | A | A |  | – | 1,248.2 | (this paper)^8^ | 1,248.2 |
| *Mesoplodon carlhubbsi* | (Moore, 1963) | 530 | 121 | A | A |  | – | 1,600.9 | (this paper)^8^ | 1,600.9 |
| *Mesoplodon carlhubbsi* | (Moore, 1963) | 439.4 | 121 | J | J | – | – | 914.9 | (this paper)^8^ | 914.9 |
| *Mesoplodon densirostris* | (Kasuya and Nishiwaki, 1971) | 356 | 83 | J | J | 397 | – | 488.2 | (this paper)^8^ | 488.2 |
| *Mesoplodon densirostris* | (Raven, 1942) | 439.42 | 108 | A | A | 397 | – | 915.0 | (this paper)^8^ | 915.0 |
| *Mesoplodon europaeus* | (Reiner et al., 1993) | 315 | 91 | N/A | J | 420 | – | 338.8 | (this paper)^8^ | 338.8 |
| *Mesoplodon europaeus* | (Santos et al., 2003) | 390 | 103.4 | N/A | J | 420 | – | 640.9 | (this paper)^8^ | 640.9 |
| *Mesoplodon ginkgodens* | (Nishiwaki et al., 1972) | 472 | 126 | A | A | – | – | 1,132.8 | (this paper)^8^ | 1,132.8 |
| *Mesoplodon ginkgodens* | (Nishiwaki et al., 1972) | 477 | 117 | A | A | – | – | 1,169.0 | (this paper)^8^ | 1,169.0 |
| *Mesoplodon ginkgodens* | (Nishiwaki et al., 1972) | 470 | 111 | J | J | – | – | 1,118.5 | (this paper)^8^ | 1,118.5 |
| *Mesoplodon grayi* | (Delhon et al., 1987) | 452.5 | 109 | A | A | – | – | 998.8 | (this paper)^8^ | 998.8 |
| *Mesoplodon grayi* | (Pinedo et al., 2001) | 446 | 95 | N/A | A | – | – | 956.5 | (this paper)^8^ | 956.5 |
| *Mesoplodon hectori* | (Cappozzo et al., 2005) | 384 | 91.6 | A | A | – | – | 611.9 | (this paper)^8^ | 611.9 |
| *Mesoplodon hectori* | (Cappozzo et al., 2005) | 394 | 99.36 | A | A | – | – | 660.7 | (this paper)^8^ | 660.7 |
| *Mesoplodon layardii* | (Pinedo et al., 2002) | 504 | 138 | A | A | – | – | 1,377.8 | (this paper)^8^ | 1,377.8 |
| *Mesoplodon mirus* | (Souza et al., 2005) | 460 | 109 | A | A | – | – | 1,049.0 | (this paper)^8^ | 1,049.0 |
| *Mesoplodon mirus* | (Raven, 1937) | 487 | 113 | A | A | – | – | 1,243.7 | (this paper)^8^ | 1,243.7 |
| *Mesoplodon mirus* | (True, 1913) | 487 | 125 | A | A | – | – | 1,243.7 | (this paper)^8^ | 1,243.7 |
| *Mesoplodon perrini* | (Dalebout et al., 2002) | 390 | 99 | A | A | – | – | 640.9 | (this paper)^8^ | 640.9 |
| *Mesoplodon perrini* | (Dalebout et al., 2002) | 443 | 97 | A | A | – | – | 937.5 | (this paper)^8^ | 937.5 |
| *Mesoplodon perrini* | (Dalebout et al., 2002) | 245 | 76 | J | J | – | – | 160.0 | (this paper)^8^ | 160.0 |
| *Mesoplodon peruvianus* | (Reyes et al., 1991) | 327 | 92 | A | A | – | – | 378.^8^ | (this paper)^8^ | 378.8 |
| *Mesoplodon peruvianus* | (Reyes et al., 1991) | 372 | 104 | A | A | – | – | 556.6 | (this paper)^8^ | 556.6 |
| *Mesoplodon peruvianus* | (Reyes et al., 1991) | 286 | 85 | J | J | – | – | 254.0 | (this paper)^8^ | 254.0 |
| *Mesoplodon peruvianus* | (Reyes et al., 1991) | 326 | 92 | J | J | – | – | 375.4 | (this paper)^8^ | 375.4 |
| *Mesoplodon stejnegeri* | (Miyazaki et al., 1987) | 396.5 | 95 | J | J |  | 544.97 | 673.3 | (this paper)^8^ | 545.0 |
| *Peponocephala electra* | (Nakajima and Nishiwaki, 1965) | 260 | 108 | A | A | 248 | – | 204.6 | (Miyazaki et al., 1998) | 204.6 |
| *Phocoena dioptrica* | (Pinedo et al., 2002) | 210 | 72 | A | A | – | – | 115.0 | (Goodall and Schiavini, 1995) based on a report of a 205 cm long specimen with a weight of 115 kg | 115.0 |
| *Phocoena phocoena* | (Viaud-Martínez et al., 2007) | 172 | 69 | N/A | A | 128 | – | 66.3 | (Lockyer, 1995) | 66.3 |
| *Phocoena phocoena* | (Viaud-Martínez et al., 2007) | 176 | 67 | N/A | A | 128 | – | 70.6 | (Lockyer, 1995) | 70.6 |
| *Phocoena phocoena* | (Viaud-Martínez et al., 2007) | 164 | 71 | N/A | A | 128 | – | 58.2 | (Lockyer, 1995) | 58.2 |
| *Phocoena phocoena* | (Viaud-Martínez et al., 2007) | 160 | 65 | N/A | A | 128 | – | 54.4 | (Lockyer, 1995) | 54.4 |
| *Phocoena phocoena* | (Viaud-Martínez et al., 2007) | 150 | 71 | N/A | A | 128 | – | 45.6 | (Lockyer, 1995) | 45.6 |
| *Phocoena phocoena* | (Viaud-Martínez et al., 2007) | 170 | 70 | N/A | A | 128 | – | 57.9 | (Lockyer, 1995) | 57.9 |
| *Phocoena phocoena* | (Viaud-Martínez et al., 2007) | 153 | 64 | N/A | A | 128 | – | 44.8 | (Lockyer, 1995) | 44.8 |
| *Phocoena phocoena* | (Viaud-Martínez et al., 2007) | 179 | 76 | N/A | A | 128 | – | 65.7 | (Lockyer, 1995) | 65.7 |
| *Phocoena phocoena* | (Viaud-Martínez et al., 2007) | 175 | 75 | N/A | A | 128 | – | 62.2 | (Lockyer, 1995) | 62.2 |
| *Phocoena phocoena* | (Viaud-Martínez et al., 2007) | 176 | 75 | N/A | A | 128 | – | 63.0 | (Lockyer, 1995) | 63.0 |
| *Phocoena phocoena* | (Viaud-Martínez et al., 2007) | 168 | 70 | N/A | A | 128 | – | 56.3 | (Lockyer, 1995) | 56.3 |
| *Phocoena phocoena* | (Viaud-Martínez et al., 2007) | 190 | 76 | N/A | A | 128 | – | 75.9 | (Lockyer, 1995) | 75.9 |
| *Physeter macrocephalus* | (Omura et al., 1962) | 1402 | 510 | J | J | 1100 | – | 26,545.0 | (Omura, 1950) | 26,545.0 |
| *Tursiops truncatus* | (Palacios et al., 2004) | 279 | 109.8 | A | A | 235 | – | 189.0 | Gihr and Pilleri 1979 in (Perrin et al., 2005) | 189.0 |
| *Ziphius cavirostris* | (Pinedo et al., 2001) | 273 | 111 | J | J | 527 | – | 221.0 | (this paper)^8^ | 221.0 |
| *Ziphius cavirostris* | (Zanelatto et al., 1995) | 620 | 175 | A | A | 527 | – | 2,556.7 | (this paper)^8^ | 2,556.7 |

S2 Table notes:

^1^ Reported specimen age: A, adult; J, juvenile; N/A, not reported.

^2^ Assigned age is either the ‘reported age’, or if not given in the published account, the specimen was considered to be mature if the reported body length was greater than published values for length at maturity.

^3^ Published length at maturity from Ridgway et al. (2016)

^4^ Published body mass of the specimen if reported in the cited reference.

^5^ Body mass predicted from published body length using the formula cited in the reference column.

^6^Reference for the body mass from body length equation.

^7^Body mass used for OCW and body mass regression, if the body mass was not published, the estimated body mass (from body length) was substituted.

^8^Equation derived from a linear regression in Excel from 53 specimens of *Hyperoodon*, *Mesoplodon*, and *Ziphius* of known length (cm) and mass (kg): log_10_ (body mass) = 2.867 X log_10_ (body length) – 4.488 (adjusted R2 = 0.93).

^9^Equation derived from a linear regression in Excel from 39 *Kogia* specimens of known length and mass: log_10_ (body mass) = 2.985 X log_10_ (body length) – 4.9265 (adjusted R2 = 0.93).

**S3 Table. Fossil cetacean brain and body mass and EQ estimates.**

| **Family** | **Species** | **Specimen numbers** | **Age (my)** | **OCW (mm)** | **OCW (reference)** | **Endocranial volume (cc)** | **Estimated brain mass (g)** | **Body mass used to calculate EQ (kg)** | **Boessenecker et al., 2017 body mass (kg)** | **EQ 0.56** | **EQ 0.75** |
| --- | --- | --- | --- | --- | --- | --- | --- | --- | --- | --- | --- |
| Basilosauridae | *Dorudon atrox* | UM 101222 | 39 | 126.0 | Uhen, (1996):p.574 | 1,173.00^8^ | 882.59 | 1,022.50 | 2,240 | 1.0480 | 0.4995 |
| Basilosauridae | *Saghacetus osiris* | BMNH 10228 | 39 | 91.8 | Kellogg, (1936):p.246-247 | 480.00^8^ | 383.44 | 378.89 | 350 | 0.7938 | 0.4569 |
| Basilosauridae | *Zygorhiza kochii* | USNM 16639 | 37 | 112.0 | Kellogg, (1936):p.246-247 | 917.00^8^ | 701.45 | 706.80 | 998 | 1.0242 | 0.5236 |
| Basilosauridae | *Zygorhiza kochii* | FMNH PM-459 | 34^5^ | 120.0 | Gingerich, (Gingerich, 2015):p. 168 | 1,189.00^8^ | 893.82 | 877.47 | 998 | 1.1562 | 0.5673 |
| Protocetidae | *Rodhocetus kasrani* | GSP-UM 3012 | 47 | 88.7 | Uhen, per. com. 2020 | 290.00^8^ | 239.62 | 340.20 | 290 | 0.5269 | 0.3095 |
| Remingtonocetidae | *Dalanistes ahmedi* | GSP-UM 3106 | 45 | 104.8 | Uhen, per. com. 2020 | 400.00^8^ | 323.46 | 573.90 | 750 | 0.5307 | 0.2823 |
| Remingtonocetidae | *Remingtonocetus harudiensis* | IITR-SB 2770 | 42^6^ | 87.0 | Bajpai et al., (2011): p.709 | 253.00^8^ | 210.97 | 320.18 | – | 0.4799 | 0.2852 |
| Aetiocetidae | *cf. Fucaia* sp*.* | USNM 256604 | 33 | 72.8 | Uhen, per. com. 2020 | 256.70 | 213.84 | 182.75 | 78.08 | 213.84 | 182.75 |
| Agorophiidae | Genus Y n. sp. | ChM PV2757 | 25 | 134.5 | Uhen, per. com. 2020 | 1,234.40 | 925.62 | 1,255.59 | 594.7 | 925.62 | 1,255.59 |
| Delphinidae | *Globicephala baereckeii* | USNM 21867 | 1 |  |  | 4,165.90 | 2,879.34 | 900.77^2^ | 900.8 | 3.6704 | 1.7920 |
| Delphinidae | *Lagenorhynchus harmatuki* | USNM 244317 | 4 |  |  | 1,129.20 | 851.80 | 98.67^2^ | 98.67 | 3.7462 | 2.7841 |
| Delphinidae | *Lagenorhynchus harmatuki* | USNM 206098 | 4 |  |  | 1,344.00 | 1,002.08 | 112.80^2^ | 112.8 | 4.0888 | 2.9625 |
| Eurhinodelphidae | *Schizodelphis longirostris* | USNM 167676 | 17 | 83.0 | Lambert, (2004): p.160 | 361.20 | 294.09 | 276.25 | 90.01 | 0.7267 | 0.4441 |
| Eurhinodelphidae | *Schizodelphis longirostris* | USNM 175379, 187211, 187212,  187213 | 14 | 82.4^7^ | Uhen, per. com. 2020 | 388.10 | 314.48 | 270.04 | 94.82 | 0.7870 | 0.4831 |
| Eurhinodelphidae | *Schizodelphis longirostris* | USNM 16118, 244413 | 15 | 77.9^7^ | Uhen, per. com. 2020 | 495.10 | 394.69 | 226.45 | 89.98 | 1.0901 | 0.6919 |
| Eurhinodelphidae | *Schizodelphis longirostris* | USNM 187306, 187627 | 16 | 81.3^7^ | Uhen, per. com. 2020 | 595.60 | 468.96 | 258.90 | 84.25 | 1.2017 | 0.7435 |
| Eurhinodelphidae | *Schizodelphis* n. sp. B. | USNM 187312 | 14 | 78.0 | Lambert, (2004): p.160 | 346.10 | 282.60 | 227.36 | 74.38 | 0.7788 | 0.4939 |
| Eurhinodelphidae | *Schizodelphis n. sp. B.* | USNM 187317, 244403, 244409 | 15 | 75.6^7^ | Lambert, (2004): p.160 and Uhen, per. com. 2020 | 478.90 | 382.62 | 206.14 | 64.9 | 1.1139 | 0.7197 |
| Eurhinodelphidae | *Schizodelphis n. sp. H.* | USNM 22804, 187314 | 14 | 72.9^7^ | Uhen, per. com. 2020 | 342.10 | 279.56 | 183.93 | 62.43 | 0.8675 | 0.5728 |
| Eurhinodelphidae | *Schizodelphis n. sp. H.* | USNM 187621 | 15 | 81.0 | Uhen, per. com. 2020 | 476.90 | 381.13 | 255.92 | 85.89 | 0.9830 | 0.6095 |
| Eurhinodelphidae | *Schizodelphis n. sp. H.* | USNM 13871, 13876 | 16 | 69.6^7^ | Uhen, per. com. 2020 | 642.70 | 503.47 | 159.07 | 57.03 | 1.6947 | 1.1502 |
| Eurhinodelphidae | *Schizodelphis sp.* | USNM 187214 | 14 | 79.0 | Back calculated^4^ | 328.00 | 268.79 | 236.63 | 78.08 | 0.7243 | 0.4559 |
| Eurhinodelphidae | *Schizodelphis sp.* | USNM 205772 | 15 | 76.0 | Back calculated^4^ | 416.20 | 335.67 | 209.58 | 67.36 | 0.9682 | 0.6236 |
| Eurhinodelphidae | *Xiphiacetus bossi* | USNM 20128 | 14 | 82.0 | Back calculated^4^ | 625.90 | 491.18 | 265.95 | 90.01 | 1.2398 | 0.7632 |
| Eurhinodelphidae | *Xiphiacetus bossi* | USNM 10714 | 18 | 89.0 | Lambert, (2005):p. 668 | 949.30 | 724.47 | 343.83 | 123 | 1.5837 | 0.9285 |
| Eurhinodelphidae | *Xiphiacetus cristatus* | USNM 13436, 13875, 167675 | 14 | 92.4 | Uhen, per. com. 2020 | 769.70 | 595.72 | 386.71 | 91.68 | 1.2193 | 0.6990 |
| Eurhinodelphidae | *Xiphiacetus cristatus* | USNM 167675 | 15 | 92.4 | Uhen, per. com. 2020 | 816.30 | 629.30 | 386.71 | 191.9 | 1.2880 | 0.7385 |
| Eurhinodelphidae | *Xiphiacetus n. sp. M.* | USNM 167622 | 14 | 75.0 | Back calculated^4^ | 531.80 | 421.92 | 201.06 | 64.04 | 1.2456 | 0.8086 |
| Eurhinodelphidae | *Xiphiacetus n. sp. V.* | USNM 21361 | 16 | 79.0 | Back calculated^4^ | 543.10 | 430.28 | 236.63 | 78.08 | 1.1595 | 0.7298 |
| Eurhinodelphidae | *Xiphiacetus n. sp. V.* | USNM 175381 | 15 | 99.0 | Back calculated^4^ | 627.20 | 492.13 | 480.08 | 184.7 | 0.8924 | 0.4910 |
| Eurhinodelphidae | *Xiphiacetus sp.* | USNM 299947 | 20 | 72.8 | Uhen, per. com. 2020 | 431.50 | 347.17 | 183.14 | 57.77 | 1.0799 | 0.7136 |
| Eurhinodelphidae | *Xiphiacetus sp.* | USNM 214767 | 14 | 84.0 | Uhen, per. com. 2020 | 574.50 | 453.44 | 286.82 | 98.67 | 1.0971 | 0.6657 |
| Eurhinodelphidae | *Xiphiacetus sp.* | USNM 171103 | 14 | 73.0 | Back calculated^4^ | 650.80 | 509.39 | 184.72 | 57.77 | 1.5769 | 1.0403 |
| Kentriodontidae | *Kentriodon n. sp. W* | USNM 317882 | 14 | – | – | 305.10 | 251.24 | 49.23^2^ | 49.23 | 1.6310 | 1.3834 |
| Kentriodontidae | *Kentriodon pernix* | USNM 10670 | 18 | – | – | 230.30 | 193.25 | 18.30^2^ | 18.3 | 2.1838 | 2.2354 |
| Kentriodontidae | *Kentriodon pernix* | USNM 25005 | 15 | – | – | 276.00 | 228.81 | 17.04^2^ | 17.04 | 2.6909 | 2.7921 |
| Kentriodontidae | *Kentriodon schneideri* | USNM 323772 | 16 | – | – | 638.00 | 500.04 | 41.65^2^ | 41.65 | 3.5645 | 3.1208 |
| Kogiidae | *Aprixokogia kelloggi* | USNM 187015 | 4 | 129.0 | Uhen, per. com. 2020 | 2,348.40 | 1,686.69 | 1,100.78 | 506.7 | 1.9217 | 0.9032 |
| Phocoenidae | *Semirostrum ceruttii* | SDSNH 65276 | 4 | – | – | 698.9^3^ | 544.43 | 103.23^2^ | 103.2 | 2.3346 | 1.7202 |
| Physeteridae | *Orycterocetus crocodilinus* | USNM 14730 | 15 | 122.0 | Kellogg (1965):p.57 | 2,188.90 | 1,579.56 | 924.14 | 422.6 | 1.9848 | 0.9643 |
| Platanistidae | *Allodelphis pratti* | YPM 13408 | 21 | 97.1 | Uhen, per. com. 2020 | 854.80 | 656.95 | 451.79 | 170.8 | 1.2325 | 0.6860 |
| Platanistidae | *Dilophodelphis fordycei* | USNM 214911 | 20 | 73.0 | Boersma et al. (2017):p:11 | 486.50 | 388.29 | 184.72 | 57.77 | 1.2020 | 0.7930 |
| Platanistidae | *Pomatodelphis? sp.* | USNM 323775 | 6 | 97.7 | Back calculated | 365.30 | 297.21 | 460.60 | 175.6 | 0.5516 | 0.3059 |
| Simocetidae | *Simocetus rayi* | USNM 256517 | 33 | 65.0 | Uhen, per. com. 2020 | 530.60 | 421.03 | 128.38 | 94.27 | 1.5979 | 1.1297 |
| Squalodontidae | *Squalodon calvertensis* | USNM 328343 | 20 | 95.0 | Uhen, per. com. 2020 | 617.20 | 484.81 | 421.86 | 157.8 | 0.9451 | 0.5330 |
| Squalodontidae | *Squalodon calvertensis* | USNM 10484 | 17 | 95.0 | Kellogg (1923) P.61 | 630.70 | 494.70 | 421.86 | 151.5 | 0.9644 | 0.5438 |
| Xenorophidae | *Albertocetus meffordorum* | CCNHM 218 | 33 | 70.7 | Boessenecker, et al. (2017):p.7 | 478.80 | 382.55 | 167.08 | 51.13 | 1.2527 | 0.8423 |
| Xenorophidae | *Xenorophus* n. sp. | ChM PV4266 | 25 | 83.6 | Uhen, per. com. 2020 | 861.40 | 661.68 | 282.56 | 96.85 | 1.6145 | 0.9825 |
| Ziphiidae | *Squaloziphius emlongi* | USNM 181528 | 23 | 108.0 | De Muizon, (1990):p.292 | 706.90 | 550.24 | 630.64 | 164.2 | 0.8564 | 0.4474 |
| Mammalodontidae | *Janjucetus hunderi* | NMV P216929 | 26.5^12^ | 109 | Occipital condyles broken, but attachment preserved, OCW estimated from Figure 1D of Fitzgerald 2006 (2006): | 708.61^9^ | 551.49 | 649.13 | - | 0.8446 | 0.4388 |
| Pelocetidae | *Parietobalaena palmeri* | USNM 205950 | 16.4^12^ | 102.8 | Measured on CT scan of the specimen available at morphosource.org | 1073.58^9^ | 812.59 | 540.58 | - | 1.3787 | 0.7417 |
| Cetotheriidae | *Piscobalaena nana* | MNHN SAS1623 | 7.5^12^ | 131 | Bouetel and de Muizon, (2006) p. 393 | 1430^9^ | 1,061.78 | 1,155.17 | - | 1.1775 | 0.5483 |
| Llanocetidae | *Llanocetus denticrenatus* | USNM 183022 | 34.7^12^ | 216 | Fordyce and Marx (2018) Table S1 | 2525^10^ | 1,804.74 | 5,540.04 | - | 0.8319 | 0.2876 |
| Aetiocetidae | *Fucaia goedertorum* | LACM 131146 | 27.9^12^ | 78 | Barnes et al., (1994): table 1 | 618.76^9^ | 485.95 | 227.36 | - | 1.3392 | 0.8493 |

S3 Table notes:

Age (my), from Boessenecker et al. (2017) unless noted otherwise; OCW (reference), indicates the source of the OCW number; Endocranial volumes from Boessenecker et al. (2017) unless indicated; Endocranial volume reduced by 20% (cc), as described in the main text, we calculate two brain masses for each basilosaurid, this column is the published endocranial volume reduced by 20% (in volume) from which we estimate an alternate brain mass and in turn alternate EQ values.

^1^ Brain mass estimate my not come from the listed specimen number, see S1 Text for details.

^2^ Body mass from Boessenecker et al. (2017), not this study, we retain their body masses for the delphinoids as explained in the main text.

^3^ Brain mass from Racicot and Rowe (2014):p.656.

^4^ Back calculated, indicates the OCW was calculated from the published body mass of Boessenecker et al. (2017) by reversing the equation used to estimate mass from OCW.

^5^ Age from range given by Gingerich (2015).

^6^ Age from Bajpai et al. (2011).

^7^ OCW is the geometric mean of the specimens listed.

^8^ Endocranial volume source listed in text Table 2.

^9^ Endocranial volume from McCurry et al. (2021).

^11^ Endocranial volume from Mitchell (1989): p.2226.

^12^ Age from McCurry et al. (2021).

**Table S4. Results of ANOVA and HSD**

| **ANOVA** |  | Df | Sum of squares | Mean of squares | F | P value |
| --- | --- | --- | --- | --- | --- | --- |
|  | Age | 2 | 0.29977 | 0.1499 | 23.8 | **0.0007564** |
|  | Residuals | 7 | 0.04412 | 0.0063 |  |  |

| **HSD** | Age groups | Oligocene | Late Eocene | Middle Eocene |
| --- | --- | --- | --- | --- |
|  | Oligocene |  |  |  |
| d | Late Eocene | 0.1211605 |  |  |
|  | Middle Eocene | **0.0006041** | **0.0090821** |  |

Table S4 – Results of ANOVA and Tukey’s Honest Significance Difference (HSD) of species mean log_10_ EQ_0.56_ scores; a); Df, degrees freedom; F, F statistic; HSD results reported as adjusted P values, bold indicates significant differences. The data included are the Middle Eocene archaeocetes (n=3, remingtonocetids and protocetids), Late Eocene basilosaurids (n=3 species, 4 individuals), and the Oligocene (n=4) odontocetes.

**References S1, S2, S3 and S4 Tables**

Allen, G.M., 1916, The Whalebone Whales of New England: Memoirs of the Boston Society of Natural History, v. 8, p. 107–322.

Arnold, P., Marsh, H., and Heinsohn, G., 1987, The occurrence of two forms of minke whales in east Australian waters with a description of external characters and skeleton of the diminutive or dwarf form: Scientific Reports of the Whales Research Institute, v. 38, p. 1–46.

Bajpai, S., Thewissen, J.G.M., and Conley, R.W., 2011, Cranial anatomy of middle Eocene *Remingtonocetus* (Cetacea, Mammalia) from Kutch, India: Journal of Paleontology, v. 85, p. 703–718.

Balensiefer, D.C., Marcondes, M.C.C., Pretto, D.J., Cypriano-Souza, A.L., and Luna, F.O., 2014, Antarctic minke whale (*Balaenoptera bonaerensis*, Burmeister, 1867) in the Tapajós River, Amazon Basin, Brazil: Aquat Mamm, v. 40, p. 201–206.

Barnes, L.G., Kimura, M., Furusawa, H., and Sawamura, H., 1994, Classification and distribution of Oligocene Aetiocetidae (Mammalia; Cetacea; Mysticeti) from western north America and Japan: Island Arc, v. 3, p. 392–431.

Boersma, A.T., McCurry, M.R., and Pyenson, N.D., 2017, A new fossil dolphin *Dilophodelphis fordycei* provides insight into the evolution of supraorbital crests in Platanistoidea (Mammalia, Cetacea): Royal Society Open Science, v. 4, p. 170022.

Boessenecker, R.W., Ahmed, E., and Geisler, J.H., 2017, New records of the dolphin *Albertocetus meffordorum* (Odontoceti: Xenorophidae) from the lower Oligocene of South Carolina: Encephalization, sensory anatomy, postcranial morphology, and ontogeny of early odontocetes: PLoS ONE, v. 12, p. e0186476.

Bouetel, V., and De Muizon, C., 2006, The anatomy and relationships of *Piscobalaena nana* (Cetacea, Mysticeti), a Cetotheriidae s.s. from the early Pliocene of Peru: Geodiversitas, v. 28, p. 319–395.

Cappozzo, H.L., Negri, M.F., Mahler, B., Lia, V. V., Martinez, P., Gianggiobe, A., and Saubidet, A., 2005, Biological data on two Hector’s beaked whales, *Mesoplodon hectori*, stranded in Buenos Aires province, Argentina: Latin American Journal of Aquatic Mammals, v. 4, p. 113–128.

Chou, W.H., 1989, First record of dwarf sperm whale (*Kogia simus*) from Taiwan: Bulletin of the National Museum of Natural Science, v. 1, p. 23–37.

Dalebout, M.L., Mead, J.G., Baker, C.S., Baker, A.N., and Van Helden, A.L., 2002, A new species of beaked whale *Mesoplodon perrini* sp. n. (Cetacea: Ziphiidae) discovered through phylogenetic analyses of mitochondrial DNA sequences: Marine Mammal Science, v. 18, p. 577–608.

Delhon, G.A., Crespo, E.A., and Pagnoni, G., 1987, Stranding of a specimen of Gray’s beaked whale at Puerto Pirámide (Chubut, Argentina) and its gonadal appraisal: Scientific Reports of the Whales Research Institute, v. 38, p. 107–115.

Fitzgerald, E.M.G., 2006, A bizarre new toothed mysticete (Cetacea) from Australia and the early evolution of baleen whales: Proceedings of the Royal Society B: Biological Sciences, v. 273, p. 2955–2963.

Fordyce, R.E., and Marx, F.G., 2018, Gigantism Precedes Filter Feeding in Baleen Whale Evolution: Current Biology, v. 28, p. 1670-1676.e2.

George, J.C., 2009, Growth, morphology and energetics of bowhead whales (*Balaena mysticetus*): University of Alaska Fairbanks, 168 p.

Gingerich, P.D., 2015, New partial skeleton and relative brain size in the late Eocene Archaeocete *Zygorhiza kochii* (Mammalia, Cetacea) from the Pachuta Marl of Alabama, with a note on contemporaneous Pontogeneus brachyspondylus: Contributions from the Museum of Paleontology, University of Michigan, v. 32, p. 161–188.

Goodall, R.N.P., and Schiavini, A.C.M., 1995, On the biology of the spectacled porpoise, *Australophocaena dioptrica*: Report of the International Whaling Commission, v. 16, p. 411–453.

Heise, K., 1997, Life history and population parameters of Pacific white-sided dolphins (*Lagenorhynchus obliquidens*): Report of the International Whaling Commission, v. 47, p. 817–825.

Kasuya, T., and Nishiwaki, M., 1971, First record of *Mesoplodon densirostris* from Formosa: Scientific Reports of the Whales Research Institute, v. 23, p. 129–137, 5 pls.

Kellogg, R., 1923, Description of two squalodonts recently discovered in the Calvert Cliffs, Maryland; and notes on the shark-toothed cetaceans: Proceedings of the United States National Museum, v. 62, p. 1–69.

Kellogg, R., 1936, A review of the Archaeoceti: Carnegie Institution of Washington Publication, v. 482, p. 1–366.

Kellogg, R., 1965, The Miocene Calvert sperm whale *Orycterocetus*: Bulletin of the United States National Museum, v. 247, p. 47–63.

Kitchener, A.C., Herman, J.S., Reid, R.J., and Anderson, N., 2012, First records of the pygmy sperm whale, *Kogia breviceps*, in Scotland: The Glasgow Naturalist, v. 25, p. 142–147.

Köppel, A., 1898, Vergleichende Bestimmungen des Innenvolumens der Rückgrat-und Schädelhöhle bei Menschen und Thieren: Archiv Für Anthropologie, v. XXV, p. 171–184.

Lambert, O., 2004, Systematic revision of the Miocene long-snouted dolphin *Eurhinodelphis longirostris* DU BUS, 1872 (Cetacea, Odontoceti, Eurhinodelphinidae): Bulletin de l’Institut Royal Des Sciences Naturelles de Belqique, Sciences de La Terre, v. 74, p. 147–174.

Lambert, O., 2005, Phylogenetic affinities of the long-snouted dolphin *Eurhinodelphis* (Cetacea, Odontoceti) from the Miocene of Antwerp, Belgium: Palaeontology, v. 48, p. 653–679.

Layne, J.N., 1965, Observations on marine mammals in Florida waters: Bulletin of the Florida State Museum, v. 9, p. 131–181.

Lockyer, C., 1995, Aspects of the biology of the harbour porpoise, *Phocoena phocoena*, from British waters: Developments in Marine Biology, v. 4, p. 443–457.

Lockyer, C.H., 1976, Body weights of some species of large whales: ICES Journal of Marine Science, v. 36, p. 259–273.

Martín, V., Tejedor, M., Pérez-Gil, M., Dalebout, M.L., Arbelo, M., and Fernández, A., 2011, A sowerby’s beaked whale (*Mesoplodon bidens*) stranded in the Canary Islands: The most southern record in the eastern North Atlantic: Aquatic Mammals, v. 37, p. 512–519.

Martins, H.R., Clarke, M.R., Reiner, F., and Santos, R.S., 1985, A pygmy sperm whale, *Kogia breviceps* (Blainville, 1838) (Cetacea: Odontoceti) stranded on Faial Island, Azores, with notes on cephalopod beaks in stomach: Arquipélago-Série Ciências Da Natureza, v. 6, p. 63–70.

McCann, C., 1975, A study of the genus *Berardius* Duvernoy: Scientific Reports of the Whales Research Institute, v. 27, p. 111–137.

McCurry, M.R., Marx, F.G., Evans, A.R., Park, T., Pyenson, N.D., Kohno, N., Castiglione, S., and Fitzgerald, E.M.G., 2021, Brain size evolution in whales and dolphins: new data from fossil mysticetes: Biological Journal of the Linnean Society,.

Meirelles, A.C.O. de, Choi, K.F., and Oliveira, M.S. de, 2011, First reported stranding of a dwarf minke whale, *Balaenoptera acutorostrata* (Lacépède, 1804), on the coast of Ceará, Northeastern Brazil: Arquivos de Ciências Do Mar, v. 44, p. 106–109.

Mitchell, E., and Kozicki, V.M., 1975, Autumn stranding of a northern bottlenose whale (*Hyperoodon ampullatus*) in the Bay of Fundy, Nova Scotia: Journal of the Fisheries Board of Canada, v. 32, p. 1019–1040.

Mitchell, E.D., 1989, A new cetacean from the late Eocene La Meseta formation Seymour Island, Antarctic peninsula: Canadian Journal of Fisheries and Aquatic Sciences, v. 46, p. 2219–2235.

Miyazaki, N., Fujise, Y., and Iwata, K., 1998, Biological analysis of a mass stranding of melon-headed whales (*Peponocephala electra*) at Aoshima, Japan: Bulletin of the National Science Museum, v. 24A, p. 31–60.

Miyazaki, N., Nakamura, I., Tanabe, S., and Tatsukawa, R., 1987, A Stranding of *Mesoplodon stejnegeri* in the Maizuru Bay Sea of Japan: Scientific Reports of the Whales Research Institute Tokyo., v. 38, p. 91–106.

Mobilio, C., 1915, Encefalo e sue parti e capacità cranica in rapporto al peso del corpo e fra loro negli Equidi: Monitore Zoologico Italiano, v. 26, p. 273–304.

Moore, J.C., 1963, Recognizing certain species of beaked whales of the Pacific Ocean: American Midland Naturalist, p. 396–428.

de Muizon, C., 1990, A new Ziphiidae (Cetacea) from the Early Miocene of Washington State (USA) and phylogenetic analysis of the major groups of odontocetes: Bulletin Du Muséum National d’histoire Naturelle. Section C, Sciences de La Terre, Paléontologie, Géologie, Minéralogie, v. 12, p. 279–326.

Nakajima, M., and Nishiwaki, M., 1965, The first occurrence of a porpoise (*Electra electra*) in Japan: Scientific Reports of the Whales Research Institute, v. 19, p. 91–104.

Nishiwaki, M., and Kasuya, T., 1970a, A Greenland right whale caught at Osaka Bay: Scientific Reports of the Whales Research Institute, v. 22, p. 45–62.

Nishiwaki, M., and Kasuya, T., 1970b, Recent record of gray whale in the adjacent waters of Japan and a consideration on its migration: Scientific Reports of the Whales Research Institute, v. 22, p. 29–37.

Nishiwaki, M., and Kasuya, T., 1971, Osteological note of an Antarctic sei whale: Sci Rep Whales Res Inst, v. 23, p. 83–89.

Nishiwaki, M., Kasuya, T., Kureha, K., and Oguro, N., 1972, Further comments on *Mesoplodon ginkgodens*: Scientific Reports of the Whales Research Institute, v. 24, p. 43–56.

Nishiwaki, M., Kasuya, T., Kamiya, T., Tobayama, T., and Nakajima, M., 1965, *Feresa attenuata* captured at the Pacific coast of Japan in 1963: Sci. Rep. Whales Res. Inst, v. 19, p. 65–90.

Ohsumi, S., Masaki, Y., and Kawamura, A., 1970, Stock of the Antarctic minke whale: Sci. Rep. Whales Res. Inst, v. 22, p. 75–125.

Omura, H., 1950, On the body weight of sperm and sei whales located in the adjacent waters of Japan: Scientific Reports of the Whales Research Institute, v. 4, p. 1–13.

Omura, H., 1958, North Pacific right whale: Scientific Reports of the Whales Research Institute, v. 13, p. 1–52.

Omura, H., 1959, Bryde’s whale from the coast of Japan: Sci. Rep. Whales Res. Inst, v. 14, p. 1–33.

Omura, H., 1975, Osteological study of the minke whale from the Antarctica: Scientific Reports of the Whales Research Institute, v. 27, p. 1–36, 16 pls.

Omura, H., and Takahashi, Y., 1981, A pygmy sperm whale stranded at Tokaimura, Ibaragi, Japan: Scientific Reports of the Whales Research Institute, Tokyo, v. 33, p. 119–124.

Omura, H., Fujino, K., and Kimura, S., 1955, Beaked whale *Berardius bairdi* of Japan, with notes on *Ziphius cavirostris*: Scientific Reports of the Whales Research Institute, v. 10, p. 89–132.

Omura, H., Ichihara, T., and Kasuya, T., 1970, Osteology of pygmy blue whale with additional information on external and other characteristics: Sci. Rep. Whales Res. Inst, v. 22, p. 1–27.

Omura, H., Nishiwaki, M., and Kasuya, T., 1971, Further studies on two skeletons of the black right whale in the North Pacific: Sci. Rpts. Whales Res. Inst., v. 23, p. 71–81.

Omura, H., Shirakihara, M., and Ito, H., 1984, A pygmy sperm whale accidentally taken by drift net in the North Pacific: Scientific Reports of the Whales Research Institute, Tokyo, v. 35, p. 183–193.

Omura, H., Nishiwaki, M., Ichihara, T., and Kasuya, T., 1962, Osteological note of a sperm whale: Scientific Reports of the Whales Research Institute, v. 16, p. 35–45.

Omura, H., Kasuya, T., Kato, H., and Wada, S., 1981, Osteological Study of the Brydes Whale *Balaenoptera edeni* from the Central South Pacific and Eastern Indian Ocean: Scientific Reports of the Whales Research Institute, v. 33, p. 1–26.

Omura, H., Ohsumi, S., Nemoto, T., Nasu, K., and Kasuya, T., 1969, Black right whales in the North Pacific: Scientific Reports of the Whales Research Institute, v. 21, p. 1–66.

Palacios, D.M., Salazar, S.K., and Day, D., 2004, Cetacean remains and strandings in the Galapagos Islands, 1923-2003: Latin American Journal of Aquatic Mammals, v. 3, p. 127–150.

Perrin, W.F., Dolar, M.L.L., Chan, C.M., and Chivers, S.J., 2005, Length-weight relationships in the spinner dolphin (*Stenella longirostris*): Marine Mammal Science, v. 21, p. 765–778.

Pike, G.C., 1953, Two records of *Berardius bairdi* from the coast of British Columbia: Journal of Mammalogy, v. 34, p. 98–104.

Pinedo, M.C., 1987, First Record of a Dwarf Sperm Whale from the Southwest Atlantic with Reference to Osteology Food Habits and Reproduction: Scientific Reports of the Whales Research Institute Tokyo, p. 171–186.

Pinedo, M.C., Lammardo, M.P., and Barreto, A.S., 2001, Review of *Ziphius cavirostris*, *Mesoplodon grayi* and *Lagenodelphis hosei* (Cetacea: Ziphiidae and Delphinidae) in Brazilian waters, with new records from southern Brazil: Atlantica, v. 23, p. 67–76.

Pinedo, M.C., Barrcto, A.S., Lammardo, M.P., and Andrade, A.L. V, 2002, Northernmost records of the spectacled porpoise, Layard’s beaked whale, Commerson’s dolphin, and Peale’s dolphin in the southwestern: Aquatic Mammals, v. 28, p. 32–37.

Racicot, R.A., and Rowe, T., 2014, Endocranial anatomy of a new fossil porpoise (Odontoceti, Phocoenidae) from the Pliocene San Diego Formation of California: Journal of Paleontology, v. 88, p. 652–663.

Raven, H.C., 1937, Notes on the taxonomy and osteology of two species of *Mesoplodon*:(*M. europaeus* Gervais, *M. mirus* True): American Museum Novitates, v. 905, p. 1–30.

Raven, H.C., 1942, On the structure of *Mesoplodon densirostris*, a rare beaked whale.: Bulletin of the American Museum of Natural History, v. 80, p. 23–50.

Reiner, F., 1986, First record of Sowerby’s beaked whale from Azores: Scientific Reports of the Whales Research Institute, v. 37, p. 103–170.

Reiner, F., Gonçalves, J., and Santos, R., 1993, Two new records of Ziphiidae (Cetacea) for the Azores with an updated checklist of cetacean species: ARQUIPÉLAGO. Ciências Biológicas e Marinhas = Life and Marine Sciences, v. 11A, p. 113–118.

Reite, M., Reite, E., Collins, D., Teale, P., Rojas, D.C., and Sandberg, E., 2010, Brain size and brain/intracranial volume ratio in major mental illness: BMC Psychiatry, v. 10, p. 79.

Reyes, J.C., Mead, J.G., and Waerebeek, K. Van, 1991, A new species of beaked whale *Mesoplodon peruvianus* sp. n.(Cetacea: Ziphiidae) from Peru: Marine Mammal Science, v. 7, p. 1–24.

Ridgway, S.H., Carlin, K.P., Van Alstyne, K.R., Hanson, A.C., and Tarpley, R.J., 2016, Comparison of dolphins’ body and brain measurements with four other groups of cetaceans reveals great diversity: Brain, Behavior and Evolution, v. 88, p. 235–257.

Röhrs, M., and Ebinger, P., 2001, Welche quantitativen Beziehungen bestehen bei Säugetieren zwischen Schädelkapazität und Hirnvolumen: Mammalian Biology, v. 66, p. 102–110.

Rosel, P.E., Wilcox, L.A., Monteiro, C., and Tumlin, M.C., 2016, First record of Antarctic minke whale, *Balaenoptera bonaerensis*, in the northern Gulf of Mexico: Marine Biodiversity Records, v. 9, p. 1–9.

Sanino, G.P., and Yanez, J.L., 1997, Estudios sobre un cachalote pigmeo, *Kogia breviceps* (De Blainville, 1838), varado en la costa de Chile: Boletín Del Museo Nacional de Historia Natural (Chile), v. 46, p. 81–93.

Santos, M.C. de O., Zampirolli, É., De, A.F.V., and Alvarenga, F.S., 2003, A Gervais’ beaked whale (*Mesoplodon europaeus*) washed ashore in southeastern Brazil: extra limital record? Aquatic Mammals, v. 29, p. 404–410.

Secchi, E.R., Barcellos, L., Zerbini, A.N., and Dalla Rosa, L., 2003, Biological observation on a dwarf minke whale (*Balaenoptera accutorostrata*), caught in southern Brazilian waters, with a new record of prey for the species: Latin American Journal of Aquatic Mammals, v. 2, p. 109–115.

Sleptsov, M., 1955, Novyi vid del’fina dal’nevostochnykh morei *Lagenorhynchus ognevi* species nova: Tr Inst Okeanol Akad Nauka USSR, v. 18, p. 60–68.

Souza, S.P., Siciliano, S., Cuenca, S., and Sanctis, B., 2005, A True’s beaked whale (*Mesoplodon mirus*) on the coast of Brazil: adding a new beaked whale species to the Western Tropical Atlantic and South America: Latin American Journal of Aquatic Mammals, v. 4, p. 129–136.

Thewissen, J.G.M., George, J.C., Rosa, C., and Kishida, T., 2011, Olfaction and brain size in the bowhead whale (*Balaena mysticetus*): Marine Mammal Science, v. 27, p. 282–294.

True, F.W., 1910, An account of the beaked whales of the family Ziphiidae in the collection of the United States National Museum, with remarks on some specimens in other American museums: Bulletin of the United States National Museum, v. 73.

True, F.W., 1913, Description of *Mesoplodon mirum*, a beaked whale recently discovered on the coast of North America: Proceedings of the United States National Museum, v. 45, p. 651–657.

Turner, W., 1882, A Specimen of Sowerby’s Whale (*Mesoplodon bidens*), Captured in Shetland: Journal of Anatomy and Physiology, v. 16, p. 458–470.

Uhen, M.D., 1996, Dorudon atrox (Mammalia, Cetacea): form, function, and phylogenetic relationships of an Archaeocete from the late middle Eocene of Egypt: University of Michigan, Ann Arbor, 608 p.

Viaud-Martínez, K.A., Vergara, M.M., Gol’din, P.E., Ridoux, V., Öztürk, A.A., Öztürk, B., Rosel, P.E., Frantzis, A., Komnenou, A., and Bohonak, A.J., 2007, Morphological and genetic differentiation of the Black Sea harbour porpoise *Phocoena phocoena*: Marine Ecology Progress Series, v. 338, p. 281–294.

Yamada, M., 1954a, An account of a rare porpoise, *Feresa* Gray from Japan: Scientific Reports of the Whales Research Institute, Tokyo, v. 9, p. 59–88.

Yamada, M., 1954b, Some remarks on the pygmy sperm whale, *Kogia*: Scientific Reports of the Whales Research Institute, Tokyo, v. 9, p. 37–61.

Zanelatto, R.C., Bittencourt, M.L., Corrêa, M.F.M., and Domit, L.G., 1995, *Ziphius cavirostris* Cuvier, 1823 (Cetacea, Ziphiidae) on the Brazilian coast, with notes on biometry: Iheringia, p. 141–147.
